# The Epstein-Barr Virus BART miRNA Cluster of the M81 Strain Modulates Multiple Functions in Primary B Cells

**DOI:** 10.1371/journal.ppat.1005344

**Published:** 2015-12-22

**Authors:** Xiaochen Lin, Ming-Han Tsai, Anatoliy Shumilov, Remy Poirey, Helmut Bannert, Jaap M. Middeldorp, Regina Feederle, Henri-Jacques Delecluse

**Affiliations:** 1 Division of pathogenesis of Virus Associated Tumors, German Cancer Research Centre (DKFZ), Heidelberg, Germany; 2 Inserm unit U1074, DKFZ, Heidelberg, Germany; 3 Department of Pathology, VU University Medical Center, Amsterdam, The Netherlands; University of North Carolina at Chapel Hill, UNITED STATES

## Abstract

The Epstein-Barr virus (EBV) is a B lymphotropic virus that infects the majority of the human population. All EBV strains transform B lymphocytes, but some strains, such as M81, also induce spontaneous virus replication. EBV encodes 22 microRNAs (miRNAs) that form a cluster within the BART region of the virus and have been previously been found to stimulate tumor cell growth. Here we describe their functions in B cells infected by M81. We found that the BART miRNAs are downregulated in replicating cells, and that exposure of B cells *in vitro* or *in vivo* in humanized mice to a BART miRNA knockout virus resulted in an increased proportion of spontaneously replicating cells, relative to wild type virus. The BART miRNAs subcluster 1, and to a lesser extent subcluster 2, prevented expression of BZLF1, the key protein for initiation of lytic replication. Thus, multiple BART miRNAs cooperate to repress lytic replication. The BART miRNAs also downregulated pro- and anti-apoptotic mediators such as caspase 3 and LMP1, and their deletion did not sensitize B-cells to apoptosis. To the contrary, the majority of humanized mice infected with the BART miRNA knockout mutant developed tumors more rapidly, probably due to enhanced LMP1 expression, although deletion of the BART miRNAs did not modify the virus transforming abilities *in vitro*. This ability to slow cell growth could be confirmed in non-humanized immunocompromized mice. Injection of resting B cells exposed to a virus that lacks the BART miRNAs resulted in accelerated tumor growth, relative to wild type controls. Therefore, we found that the M81 BART miRNAs do not enhance B-cell tumorigenesis but rather repress it. The repressive effects of the BART miRNAs on potentially pathogenic viral functions in infected B cells are likely to facilitate long-term persistence of the virus in the infected host.

## Author Summary

The Epstein-Barr virus (EBV) infects more than 90% of the human adult population. Although EBV usually causes an asymptomatic infection, it is oncogenic in a small proportion of infected individuals. EBV produces a large number of microRNAs, a type of RNA that controls the production of their proteins though multiple mechanisms. We addressed the role played by the BART microRNAs, a subgroup of the EBV microRNAs, by generating a virus that lacks them and by comparing the characteristics of this modified virus with those of the unmodified virus. We found that the BART microRNAs cooperate to curb EBV multiplication, both in infected cells and in humanized mice. Furthermore, the BART miRNAs did not potentiate EBV’s ability to form tumors in different types of mice, some of which are unable to mount an immune reaction against the virus, as could have been expected from the literature. This can be explained at the molecular level by the ability of the BART microRNAs to downregulate the synthesis of multiple cellular and viral proteins, among which caspase 3 and LMP1, two essential modulator of cell death and cell proliferation, are likely to play an important role in the outcome of the virus infection. Thus, the BART microRNAs negatively impact on two essential viral functions, probably to maintain a balance between the virus and its host.

## Introduction

The Epstein-Barr virus (EBV) is a strongly B lymphotropic virus that infects the majority of the world human population and is associated with the development of malignant tumors, mainly lymphomas and carcinomas of the nasopharynx (NPC) and of the stomach [1]. Shortly after infection, B cells start to divide and generate continuously growing cell lines, commonly termed lymphoblastoid cell lines (LCLs) [2]. Infected cells express a set of latent proteins ascribed to subfamilies known as Epstein-Barr virus nuclear antigens (EBNA) and latent membrane proteins (LMP), most of which are essential or strongly potentiate the B cell transformation process [1]. EBV also encodes 44 miRNAs that are divided into two clusters located around the BHRF1 gene (BHRF1 miRNAs) or within the introns of the BART gene (BART miRNAs) [3–5]. Viruses devoid of the BHRF1 miRNA locus are less transforming than their wild type counterparts [6–9]. Indeed, a recombinant virus that lacks this cluster retains only 1/20^th^ of the wild type transforming capacity [8].

The BART miRNAs are present in all EBV-infected cells, but their expression level is up to hundred times higher in epithelial cells than in infected B lymphocytes, suggesting that they exert their main function in the former type of cells [5,10]. EBV-associated carcinomas produce only a restricted number of latent proteins but also the BART miRNAs, making these non-coding RNAs prime suspects in the transformation process [11]. Indeed, miR-BART9 and miR-BART7-3p have been found to promote metastasis of NPC cells [12,13]. Reciprocally, anti-miR-BART7-3p reduced tumor growth in an animal model [13]. In the same vein, miR-BART3* was found to target the tumor suppressor gene DICE1 in NPC cells [14]. In the primary B cell system, Vereide et al. used a B95-8 EBV strain virus with a reconstituted BART locus to show that the BART miRNAs also improve the transforming abilities of the virus [9].

The BART miRNAs have been found to regulate apoptosis by targeting pro-apoptotic proteins such as PUMA and BIM in epithelial cells [15,16]. A photoactivatable ribonucleoside-enhanced crosslinking and immunoprecipitation (PAR-CLIP) screening of the EBV-positive NPC cell line C666 revealed that the antiapoptotic properties of the BART miRNAs can be ascribed to the direct targeting of 10 pro-apoptotic proteins in these cells [17]. The BART miRNAs have also been suggested to act as repressors of EBV lytic replication in B cell or epithelial cell lines induced with drugs such as TPA. MiR-BART6, through its ability to target DICER, and miR-BART20-5p through targeting of BZLF1, control entry into the EBV lytic replication phase [18,19]. MiR-BART18-5p also controls the onset of replication in anti-Ig-treated Akata Burkitt’s lymphoma cell lines and in LCLs induced by TPA through its ability to target the expression of MAP3K2 [20]. This is in agreement with the view that EBV-infected B cells hardly replicate the virus and are mainly latent, whilst infected epithelial cells are the main sites of replication [2]. These data have frequently been collated in tumor cells or in LCLs infected by viruses that carry a partial deletion of the BART miRNAs and might not extend to strains with an intact locus, in particular *in vivo*. Furthermore, the view that LCLs are primarily latent and must be stimulated to produce virus is restricted to viral strains close to B95-8. We have recently shown that M81, a viral strain that carries a high degree of homology with viruses found in NPC and that infects a substantial proportion of the Chinese population, induces a high degree of spontaneous virus replication upon infection of primary B cells [21]. Importantly, this virus carries an intact BART locus. Both features render M81 a suitable experimental system to study the function of the BART locus in infected primary B cells. Here we report the phenotypic traits of a set of viruses that are devoid of different subsets of the BART miRNA cluster in the fully permissive M81-based replication system.

## Results

### The BART miRNAs locus represses lytic replication in transformed B cell lines

**Fig 1:**
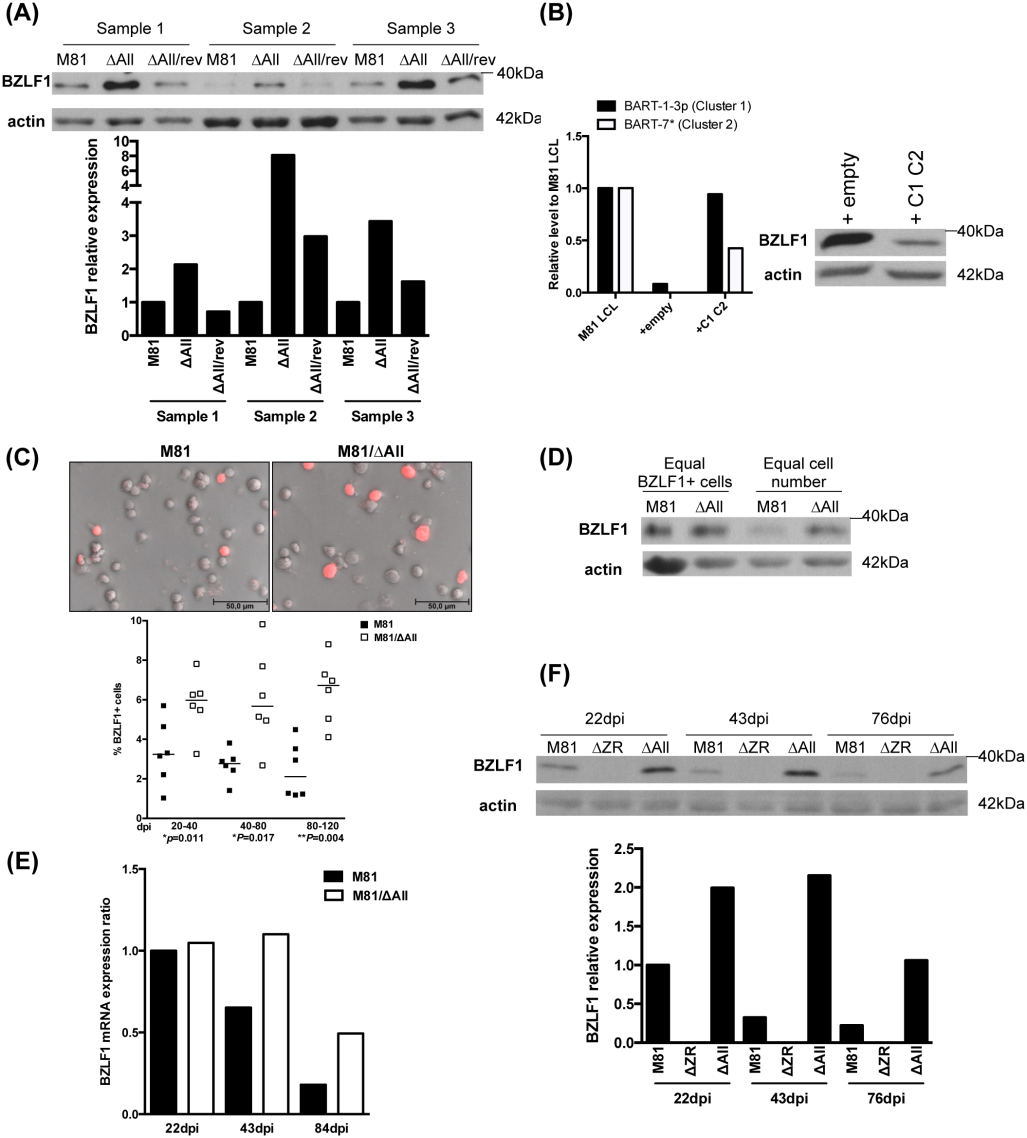
The deletion of BART miRNAs enhances BZLF1 expression in infected B cells. (A) We performed immunoblot analyses on LCLs transformed with M81, M81/ΔAll or its revertant with antibodies specific for BZLF1 and actin. The upper picture shows the results of this assay for three different donors. The relative intensity of the signals were quantified using the ImageJ software and are given as a graph of bars. (B) A LCL transformed by M81/ΔAll was stably transfected with a plasmid that encodes a truncated form of NGFR and both BART-miRNA clusters (C1C2) or with a plasmid that encodes NGFR only (empty). After 30 days, the NGFR-positive cells were purified with a specific antibody. We determined the BART-miRNA expression in these cells relative to M81 LCL (left panel) and their BZLF1 protein expression (right panel). (C) This figure depicts the BZLF1 expression pattern in LCLs transformed by M81 or M81/ΔAll as determined by immunofluorescence staining. One staining example is shown in the top pictures, whilst the percentage of BZLF1-positive cells in LCLs from multiple B-cell donors at different days post infection (dpi) is given in the scatter plot underneath. The p values obtained from paired t tests performed with LCLs infected by the two types of virus are given. Both pictures were taken at the same magnification and replicating cells appear larger, presumably as the result of cytopathic effects induced by the lytic replication. (D) This western blot analysis was performed with protein lysates from LCLs generated with M81 or M81/ΔAll that contain the same number of BZLF1-positive cells and stained with a BZLF1-specific and an actin-specific antibody. (E) We used qPCR with a BZLF1-specfic Taqman probe to determine the BZLF1 mRNA levels in LCLs transformed with M81 and M81/ΔAll. The graph of bars shows the ratio between the expression levels in the 2 types of LCLs at different time points post-infection. (F) This immunoblot shows the variation of BZLF1 protein expression in B cells infected with wild type virus or with the BART miRNA knockout mutant over a period of 76 days. Actin staining was used as a loading control. An LCL generated with the same B cells and a virus depleted with lytic transactivators BZLF1 and BRLF1 (M81ΔZR) served as a negative control. Please also see S1, S2 and S3 Figs.

We set out to determine the role of the BART miRNAs by infecting B cells with a virus devoid of this locus. To this end, we sequentially deleted the BART subcluster 1 (M81/ΔC1), subcluster 2 (M81/ΔC1C2) and miR-BART-2 (M81/ΔAll) (S1 Fig). Importantly, this mutant retains intact RPMS1, LF1 and LF2 exons. We then generated a revertant virus by reintroducing the complete BART locus in M81/ΔAll (M81/ΔAll/rev). We also individually deleted the subcluster 2 or miR-BART2 in the wild type genome to generate M81/ΔC2 and M81/Δb2 (S1 Fig). All these recombinants were stably introduced into 293 cells to generate producer cells lines that carry intact copies of the mutants, and were accordingly termed 293/M81/ΔAll et cetera (S1 Fig). We began by infecting B cells isolated from the peripheral blood with M81, M81/ΔAll and M81/ΔAll/rev to generate a panel of lymphoblastoid cell lines (LCLs). We measured BART miRNA expression in these cells for miR-BART1-3p, miR-BART7* and miR-BART2-5p that are expressed at good levels in these LCLs and are representative of each BART subcluster [22]. This confirmed that the cells infected by the mutants did not express the miRNAs that had been deleted (S2A Fig). Although the construction of the ΔAll mutant left the LF1, LF2 and LF3 genes intact, we also quantified expression of the BART mRNA by RT-PCR in LCLs generated with M81/ΔAll or wild type M81. This assay revealed that the transcript is produced in cells infected by the ΔAll mutant, on average at marginally higher levels relative to wild type (S2B Fig). However, the difference was not statistically significant (S2B Fig). We then assessed lytic replication in the LCL panel as we previously reported that M81 spontaneously replicates in B cells [21]. We first gauged BZLF1 by western blot in 18 donors (Fig 1A and S3 Fig). These assays revealed that expression of this protein is enhanced by an average 3.4 fold in the absence of the BART miRNAs, compared to LCLs infected with wild type EBV or the M81/ΔAll revertant. In two of these cases (samples F and I), the BZLF1 expression was very close or even higher in the LCL infected by the wild type virus (S3A Fig). We then attempted to complement the phenotype of LCLs transformed by M81/ΔAll by transfecting them with a plasmid that encodes all BART miRNAs with the exception of miR-BART-b2, as well as a truncated nerve growth factor receptor (NGFR) that is expressed at the surface of transfected cells, or with a control vector. We first used qPCR to confirm that NGFR antibody-purified cells transfected with the BART miRNAs express them, as shown in Fig 1B, and then performed a western blot with the same cells. Cells transfected with the BART expression vector exhibited lower levels of BZLF1 protein than those transfected with control plasmids, confirming that expression of the BZLF1 protein is modulated by the BART miRNAs.

We then performed immunofluorescence stains (IF) with the same antibody at different time points that showed that the number of BZLF1-positive cells was, on average, 2 to 3 times higher in cells infected with the BART miRNA KO mutant, relative to wild type controls (p<0.02) (Fig 1C). However, the intensity of the staining at the single cell level was not significantly stronger in BZLF1-positive cells. To confirm this observation, we performed a western blot with protein extracts from LCLs infected with the M81/ΔAll mutant or wild type virus, normalized by the number of BZLF1-positive cells in the LCL infected by the wild type control, as determined by IF (Fig 1D). Thus, these samples contained approximately the same number of BZLF1-positive B cells. Both samples generated BZLF1 signals of similar intensity, confirming that the replicating cells from LCLs infected by wild type or ΔAll mutants produce similar amounts of the BZLF1 protein. A RT-qPCR with BZLF1-specific primers performed on the LCL panel revealed that mRNA BZLF1 expression was higher in the LCLs infected with the miRNA mutant, particularly 40 days after infection but did not exceed a factor of 2.5 over time (Fig 1E). Taking into account that excision of the BART miRNAs results in an increase of the number of BZLF1-positive cells but not in an increase in protein expression at the single cell level, the likeliest explanation for the enhanced BZLF1 mRNA production is that an increased number of cell produced it, although a direct effect of the BART miRNAs on RNA stability could have contributed to this process. Altogether, we conclude that the BART miRNAs contribute to the repression of spontaneous reactivation of the lytic cycle in infected cells, but that their absence does not substantially increase BZLF1 protein expression in the individual replicating cells.

**Fig 2:**
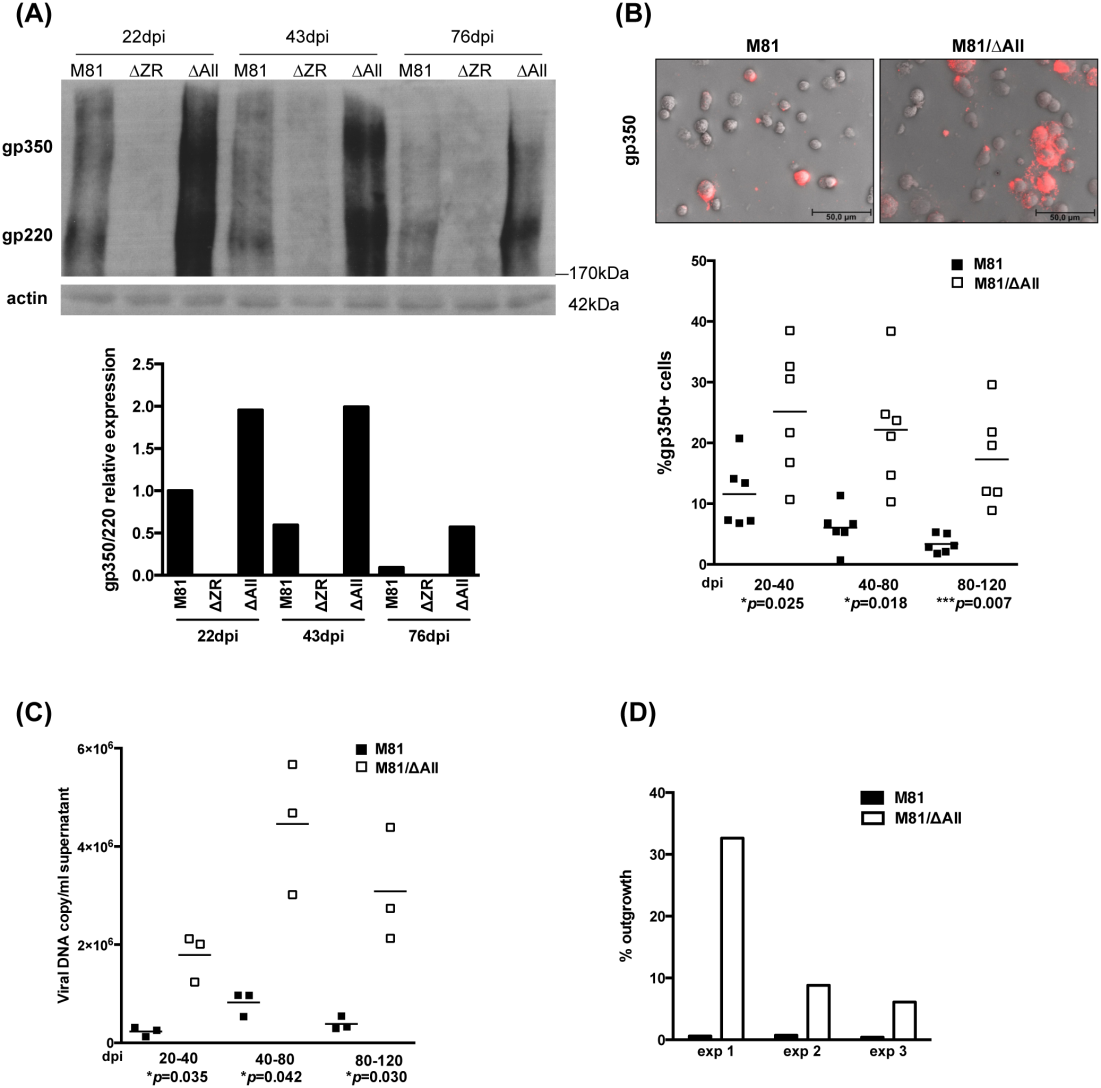
The deletion of BART miRNAs enhances virus production in infected B cells. (A) This figure shows a western blot analysis performed a different time points on B cells from the same donor transformed with M81, M81/ΔAll or ΔZR with antibodies specific for gp350 and actin. The upper picture shows expression of gp350 and of its alternative spliced form gp220 in these LCLs. The relative intensity of the signals was quantified using the ImageJ software and is depicted in a graph of bars. (B) We generated LCLs by exposing B cells from 6 different donors to M81 or M81/ΔAll. These cells were immunostained with antibodies specific for gp350 as exemplified in the top pictures. The adjacent scatter plot shows the percentage of gp350-positive cells, including cells producing gp350 and B cells covered by virions, in these LCLs at different days post infection. The figure also shows the p values obtained from paired t tests performed with the two types of LCLs. (C) We quantified the EBV DNA load in supernatants from three couple of LCLs obtained by infection with M81 or M81/ΔAll by qPCR and show the results in this scatterplot. The p values of paired t tests performed with the different types of supernatants are indicated. (D) This graph gives the result of B-cell transformation assays that were performed by exposing primary B cells to supernatants from three different LCLs obtained with M81 or M81/ΔAll virions.

We then studied expression of the BZLF1 protein over time. B cells infected by a virus that lacks BZLF1 and BRLF1 (ΔZR) served as a negative control [21]. We performed immunoblots at day 22, 43 and 84 dpi that showed an overall decrease in the expression of this protein. However, the LCLs infected with the M81/ΔAll remained longer strongly BZLF1-positive (Fig 1F). We assessed the consequences of increased BZLF1 expression by staining the infected LCLs with antibodies against gp350. Both the western blot and the IF stains showed a higher number of gp350-positive cells in cells infected with the ΔAll mutant than in those infected with the wild type virus (Fig 2A and 2B). It is important to note that the large majority of the viruses that are produced by the replicating cells bind to their neighbor B cells, some of which become covered with gp350-positive signals, making it difficult to distinguish them from producer cells [21]. Thus, the results presented here include both types of gp350-positive cells. We also measured the viral copy numbers in supernatants from LCLs by qPCR and found them clearly increased in those from LCLs generated with M81/ΔAll (Fig 2C). We then infected primary B cells with these LCL supernatants at low cell density to prove that the increased viral titers were a consequence of enhanced virus production. Indeed, this assay showed an increase in the number of outgrowing clones after treatment with supernatants from the M81/ΔAll LCL, although the efficiency of transformation widely varied between different supernatants (Fig 2D).

### Deletion of the BART miRNAs reduces recruitment of BZLF1 mRNA to the RISC

**Fig 3:**
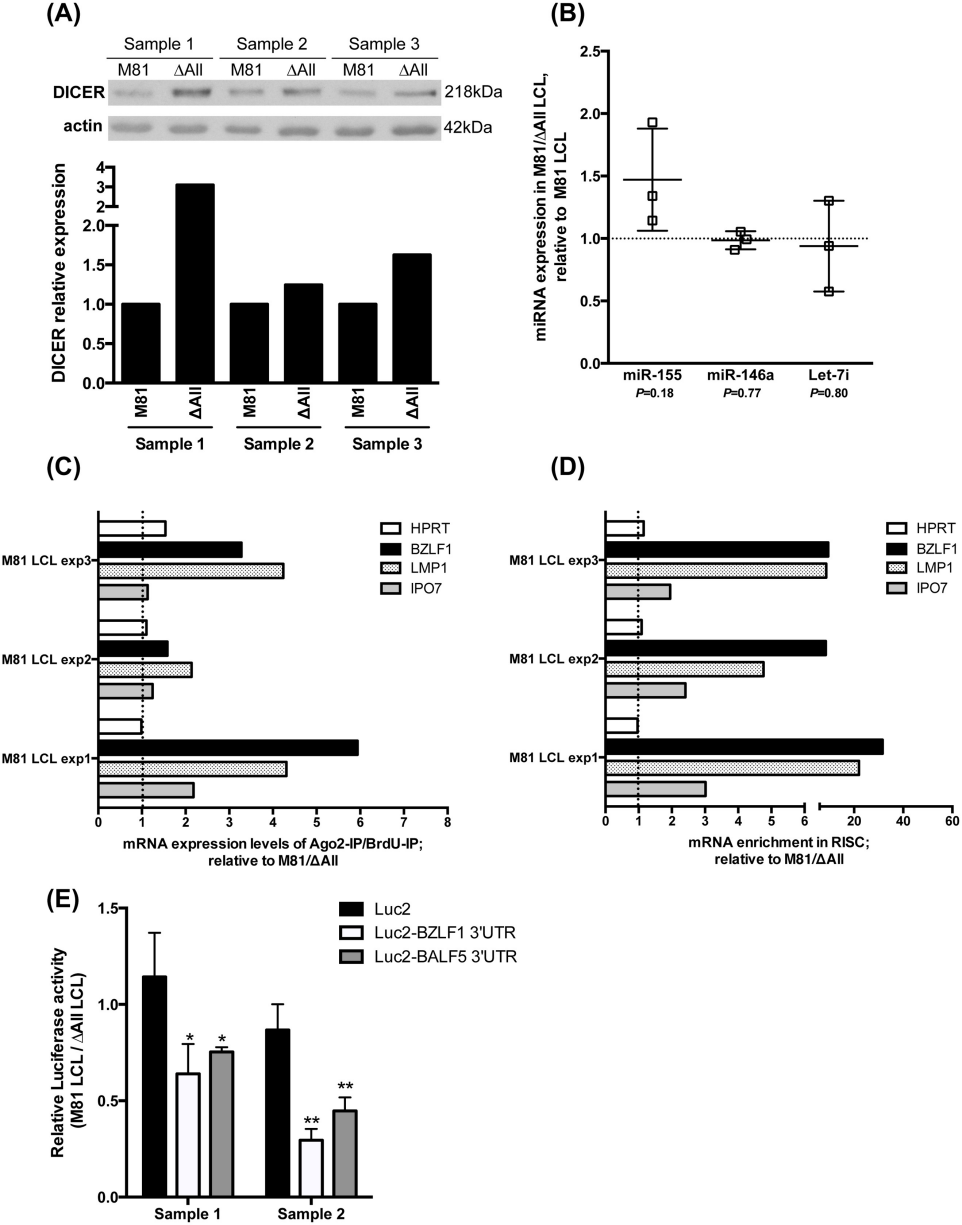
The miR-BART control expression of DICER and recruit BZLF1 mRNAs to the RISC. (A) LCLs generated with B cells from independent donors exposed to M81 and M81/ΔAll were subjected to western blot analysis with an antibody specific for DICER. Actin was used as a loading control. The intensity of the observed signals was quantified with the ImageJ software. The results of this assay are shown in a graph of bars and are given relative to the expression in LCLs transformed by wild type M81. (B) We used stem loop qPCR to gauge the expression levels of cellular miRNAs in LCLs transformed by M81 or M81/ΔAll. (C and D) RISC immunoprecipitation in LCLs generated with M81 and M81/ΔAll. We measured the expression levels of the GADPH, HPRT, BZLF1, LMP1 and IPO7 mRNAs in untreated cells or after immunoprecipitation with an anti-Ago2 antibody or with a BrdU-specific antibody. The expression of HPRT, BZLF1, LMP1 and IPO7 mRNAs were first normalized to GADPH levels. Fig 3C shows the ratio of the values obtained after immunoprecipitation with the anti-Ago2 antibody with those obtained with a BrdU-specific antibody in three experiments and Fig 3D show the values of the same experiment normalized to the mRNA expression levels in untreated total mRNAs. (E) Luciferase activity assays were performed in triplicate for 2 sets of independent LCLs generated with M81 or M81/ΔAll. We cotransfected a plasmid that encodes the rat-CD2 protein together with luciferase expression plasmids. The latter included the unmodified luciferase vector (Luc2) or a luciferase expression plasmid fused with the 3’UTR of BZLF1 (Luc2-BZLF1 3UTR) or BALF5 (Luc2-BALF5 3’UTR). We performed luciferase assays 48 hours post-electroporation and the recorded values were normalized to the percentage of CD2-positive cells that had been determined by immunofluorescence staining. The graph shows the ratios of luciferase activity between M81 and M81/ΔAll LCLs for each transfected plasmid. Error bars indicate standard deviation for the values obtained in the three technical replicates. We show the results of statistical analyses performed by two-tailed student t-test (* indicates p<0.05 and ** indicates p<0.01).

MiRNAs typically modulate expression of target genes by forming the RNA-induced silencing complex (RISC) that includes miRNAs, their mRNA targets and a member of the Argonaute family of proteins. Therefore, we tested whether the deletion of the BART miRNAs modifies the recruitment of BZLF1 mRNA to the RISC. We began by measuring the expression levels of DICER in cells lacking the BART miRNAs, as this protein has been suggested as a target of the BART miRNAs [19]. A western blot performed on three pairs of LCLs generated from three different blood donors and infected with either M81 or M81/ΔAll showed an increase in DICER protein expression (Fig 3A). However, we gauged the expression levels of three cellular miRNAs expressed in B cells but could not identify any differences between LCLs transformed with wild type EBV or with the ΔAll mutant (Fig 3B). We conclude that the impact of the BART miRNAs on DICER in infected B cells has no generalized and pronounced functional consequences. We performed RISC immunoprecipitations in couples of LCLs infected with wild type or ΔAll mutant using an antibody directed against Ago2 (S4A Fig) and measured mRNA expression by qPCR in the precipitates. We assessed the efficacy of this protocol by comparing expression of the EBV miR-BHRF1 miRNAs in the Ago2 antibody precipitate with the expression in untreated LCLs. This assay showed more than 10000-fold enrichment of this miRNA after immunoprecipitation (S4B Fig). An immunoprecipitation with an antibody directed against BrdU performed in parallel measured the non-specific background mRNA recovery. We measured the expression levels of GAPDH, HPRT, IPO7, LMP1 and BZLF1 mRNAs in the RISC of LCLs transformed with M81 or M81/ΔAll. GAPDH expression levels were used to normalize for mRNA recovery, HPRT has previously found not to be recruited in the RISC by BARTs, whereas the IPO7 mRNA is a previously validated BART target [9,23–25]. We also quantified expression of these mRNAs in infected cells. Comparison with the expression levels obtained after immunoprecipitation determines the efficacy of recruitment to the RISC. A prerequisite for this analysis is that the number of mRNA molecules than can be recruited to the RISC does not vary too much between the cells, so as to exclude saturation effects. However, we have seen that the increase in BZLF1 protein expression results from an increased number of replicating cells that all produce the protein at approximately the same level. Thus, it is unlikely that the BZLF1 mRNA expression level and recruitment to the RISC will vary significantly between different replicating cells.

The results of these experiments are given in Fig 3C and 3D, respectively. They first show the relative amounts of mRNAs in the RISC after subtraction of the background generated by the BrdU antibody. This experiment shows that BZLF1, LMP1, and to a lesser extent IPO7 mRNAs are more abundant in the RISC of LCLs infected with M81 wild type virus. In contrast, the levels of HPRT mRNAs in the RISC were similar in LCLs infected with either type of virus (Fig 3C). We then calculated the level of enrichment of these mRNAs into the RISC by comparing expression levels after Ago2 immunoprecipitation or in untreated cells (Fig 3D). This figure clearly shows that the relative recruitment in the LCLs infected with wild type viruses was more efficient for BZLF1, LMP1 and IPO7 than for HPRT. We conclude that the BART miRNAs recruit the first three mRNAs to the RISC. However, these genes are still detectable in the RISC of LCLs infected with the ΔAll mutant. Therefore, other miRNAs, presumably miRNAs of cellular origin, recruit these mRNAs to the RISC. We wished to confirm these results by performing luciferase reporter assays. To this end, we constructed a pGL4.5-based luciferase reporter plasmid that carries the BZLF1 3’UTR. We also fused the luciferase gene to the BALF5 3’UTR that has previously been reported to be directly targeted by BART-2 miRNA [26]. The luciferase expression plasmid devoid of 3’UTR was used as a negative control. These plasmids were cotransfected with a rat-CD2 expression plasmid into two independent sets of LCLs generated with either of M81 and M81/ΔAll to determine the transfection efficiency and allow normalization. We measured the luciferase activity in transfected cells and found that the luciferase activity for both plasmids carrying the BZLF1 or the BALF5 3’UTR was reduced by 40 to 60% in the wild type LCL, relative to the LCL generated with the ΔAll mutant (Fig 3E), a result previously reported for BALF5 [26]. The control plasmid pGL4.5 showed no major difference in expression (Fig 3E). These results suggest that M81 LCLs recruit more BZLF1 mRNA to the RISC than M81/ΔAll LCLs by targeting the BZLF1 3’UTR. However, the low transfection rates achieved in LCLs (1 to 2%) indicate that we should exercise caution when interpreting these results

### Lytically replicating cells express lower levels of EBV miRNAs than non-replicating counterparts

**Fig 4:**
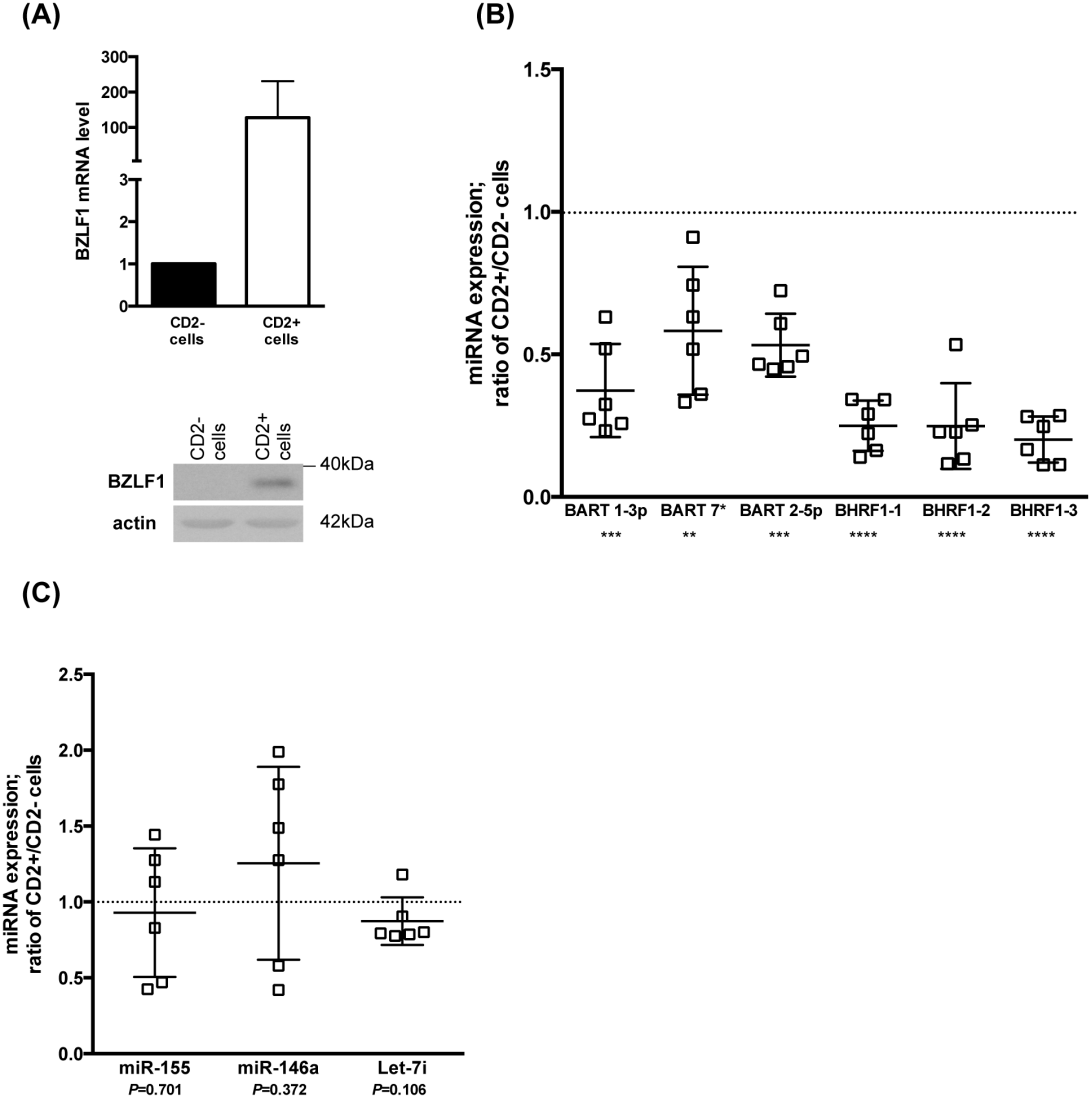
Replicating cells produce lower levels of EBV viral miRNAs than non-replicating counterparts. (A) CD2-positive cells were isolated from LCLs generated with a M81 mutant that expresses a truncated form of rat CD2 behind an EA-D-responsive promoter. CD2-positive or CD2-negative cell populations were submitted to RT-qPCR to assess BZLF1 mRNA expression (top graph) and to a western blot analysis with a BZLF1-specific antibody (Bottom picture). (B) The scatter plot shows expression of 6 viral microRNAs extracted from CD2-positive or CD2-negative cell populations obtained from 6 different LCLs generated with the CD2-expressing virus. (C) The experiment described in (B) was repeated with 3 cellular miRNAs expressed in EBV-transformed B cells. P values lower than 0.05, 0.01, 0.001, and 0.0001 obtained after paired t-student tests are indicated as *, **, ***, and **** in the figure.

The data gathered so far indicated that the BART miRNAs repress lytic replication in primary B cells. However, B cells infected with wild type EBV undergo lytic replication, although these cells can express the BART miRNAs. This paradox could be resolved if the expression levels of the BART miRNAs differed in replicating and non-replicating cells. To address this question, we constructed a virus that encodes a truncated version of the CD2 molecule, whose expression is driven by the viral EA-D promoter. Thus, infected B cells undergoing lytic replication express CD2 at their cell surface and can be immunocaptured by a specific antibody. We quantified BZLF1 expression in the CD2-positive and CD2-negative populations using quantitative RT-PCR (Fig 4A). As expected, we found that only CD2-positive cells produced BZLF1 at the RNA and protein level. This implies that cells that expressed the BZLF1 mRNA also expressed the BZLF1 protein, ie these mRNAs are not subjected to massive miRNA interference. We then quantified the expression profile of some viral and cellular miRNAs in these 2 cell populations. We found that replicating cells expressed approximately 2 times less BART miRNAs and 3 times less BHRF1 miRNAs than non-replicating cells (Fig 4B). Thus, there is an inverse relationship between EBV miRNA and BZLF1 expression. However, the cellular miRNAs were expressed at the same level irrespective of the replication status of the infected cells, suggesting that these cells did not globally downregulate miRNA synthesis (Fig 4C).

### The BART cluster 1 plays a predominant role but cluster 2 is also involved in the control of lytic replication

**Fig 5:**
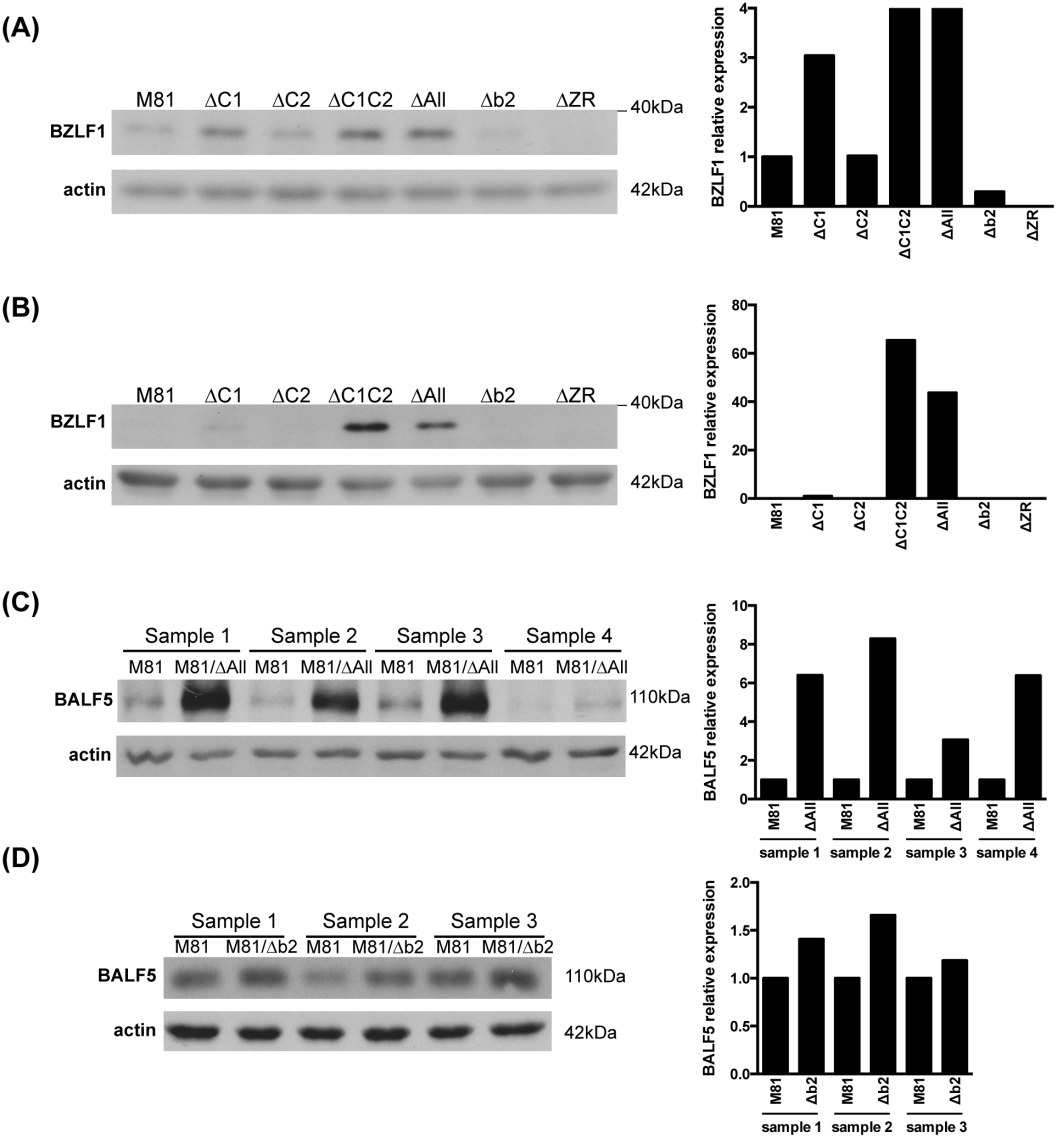
The miRNA subcluster1 is mainly but not exclusively responsible for the control of BZLF1 expression. The figure shows Western blot analyses of LCLs generated with M81, M81/ΔAll, M81/ΔC1, M81/ΔC2, M81/ΔC1C2, M81/Δb2, M81/ΔZR with a BZLF1-specific antibody. The LCLs were stained at 42 (A) and 101 (B) days post-infection. The relative intensity of the signals were quantified using the ImageJ software and are also displayed as a graph of bars. One more sample is shown in S5 Fig. (C) and (D) We determined BALF5 expression level in multiple LCLs transformed with M81 and M81/ΔAll (C) and M81/Δb2 by western blot (D), and depict the results as a graph of bars after Image J quantification. All LCLs shown in (C) and (D) were investigated between 40–43 days post infection.

Altogether, the previous results demonstrated that the BART miRNA repress spontaneous expression of BZLF1 *in vitro* and that their deletion enhances full productive lytic replication with virion production. However, the BART cluster is very large and we wished to learn the respective contribution of its subclusters. Therefore, we quantified BZLF1 expression in LCLs transformed with M81/ΔC1, M81/ΔC2 and M81/Δb2 from 2 independent blood samples at two different time points (Fig 5A, 5B and S5 Fig). These experiments showed that BZLF1 expression is higher in LCLs obtained by infection with M81/ΔC1 than in controls. Although we found no evidence for increased BZLF1 expression in M81/ΔC2, both M81/ΔC1 and M81/ΔC2 LCLs expressed BZLF1 less strongly than M81/ΔAll and M81/ΔC1C2, and this effect remained visible after 101 days of culture. LCLs infected with M81/Δb2 were indistinguishable from the wild type controls in terms of BZLF1 expression, although this miRNA has been suggested to control the onset of lytic replication [26]. However, we measured the expression of BALF5 in LCLs infected with M81/ΔAll or M81/Δb2 or wild type controls and found that the expression of BALF5 is indeed increased in LCLs generated with the mutant, particularly in those that carry M81/ΔAll (Fig 5C and 5D). However, the increased expression of BALF5 in the LCLs infected with the M81/Δb2 virus does not result from an increased replication in these cells as shown by the unchanged BZLF1 expression in LCLs generated by a virus that lacks miR-BART-2 (Fig 5A and 5B).

### The BART miRNA cluster regulates lytic replication and cell growth *in vivo*

**Fig 6:**
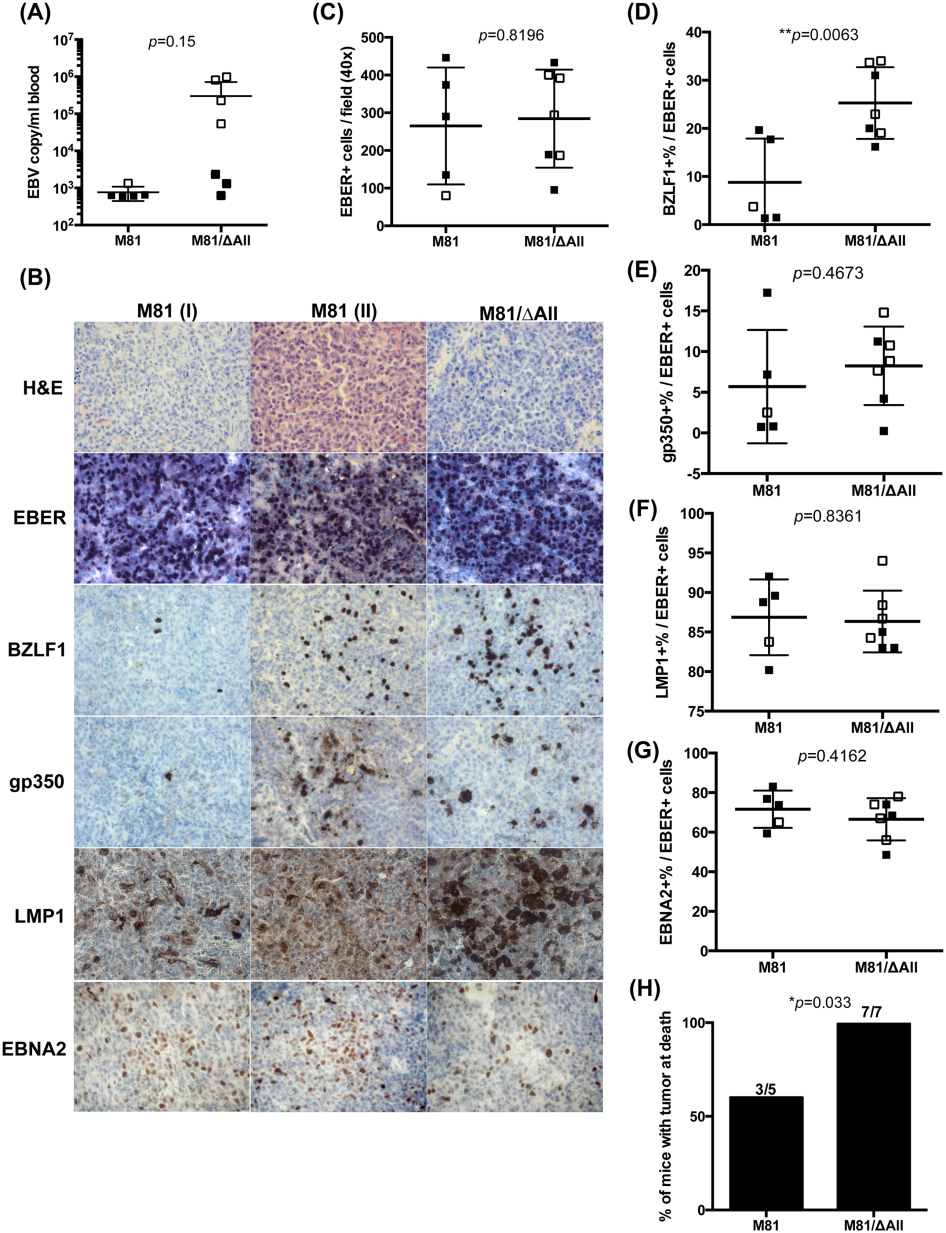
Deletion of the BART miRNAs enhances spontaneous lytic replication and tumor progression in the humanized mice model. Viral titers in peripheral blood of infected mice were determined by quantitative PCR at (A) 5 weeks post-infection. (B) The pictures show tumors that developed in the spleen. Continuous tissue sections were stained with hematoxylin and eosin (H&E), immunostained with antibodies specific for BZLF1, gp350, LMP1, EBNA2, or subjected to an *in situ* hybridization with an EBER-specific probe. Among five M81-Wt-infected mice, 3 mice (referred to as group I) had very few whilst the other 2 mice (referred to as group II) exhibited a higher percentage of BZLF1-positive cells. (C) The number of EBER positive cells per 0.04μm^2^ (surface of the field at high magnification) is given in this boxplot. (D-G) The boxplots display the ratio between (D) BZLF1-, (E) gp350-, (F) LMP1-, or (G) EBNA2-positive cells versus EBER-positive cells. The data collected from the mice euthanized at week 5 are shown as open squares. (H) This graph shows the tumor incidence for humanized mice investigated in this study. We used a one-tailed Chi-square analysis in figure H and two-tailed unpaired student t test for all other results.

We wished to complete the phenotypic analysis of the M81/ΔAll mutant by infecting humanized mice. We infected 7 mice intraperitoneally with the mutant and 5 with its wild type counterparts and measured blood viral titers 5 weeks post-infection (Fig 6A). The titers were much higher in 4 out of 7 mice infected with M81/ΔAll than in the positive controls at early time point (Fig 6A). Furthermore, animals infected with the mutant showed signs of wasting (loss of weight, apathy, food refusal, ruffled hair) and four of them had to be euthanized approximately at week 6, 2 weeks before the planned termination of the experiment. This phenomenon was not seen in mice infected with wild type virus and is statistically significant (0/5 in M81-infected versus 4/7 in M81/ΔAll-infected mice; p = 0.019 by one-tailed Chi-square test). To allow comparison with wild type infected mice, we euthanized one of these mice at the same time. Whilst gross examination of the organs showed large neoplastic nodules in the spleen of infected with the M81/ΔAll mutant, there were only a few interspersed EBER-positive cells in the spleen of the animal infected with wild type virus. The remaining mice survived until week 8 without signs of animal suffering at which time they were euthanized. Both mice infected with wild type M81 and those infected with the ΔAll mutant showed tumors in the spleen or tumors in the pancreas for 3 animals (Fig 6B and Table 1). Although the mice were not all investigated at the same time, we found that 3 out of 5 mice infected with wild type M81 and 7 out of 7 mice infected with M81/ΔAll had macroscopic tumors and the difference between the 2 groups of animals is statistically significant (Fig 6H and Table 1). Animals with tumors in the pancreas tended to have lower virus titers than those with tumors in the spleen, possibly because the tumor cells in this case have more restricted access to the blood circulation. Histological examination of the above-described neoplastic infiltrates readily confirmed the presence of activated lymphoid cells that proved to be EBER-positive (Fig 6B). We also found histological evidence of EBV-positive diffuse B cell infiltration of variable intensity after infection with M81/ΔAll and wild type EBV in other organs such as the liver, or the pancreas (Fig 6B and Table 1). The density of EBER-positive cells in these organs was similar in mice infected with wild type or with the mutant, although there was great variation in the concentration of EBV-positive cells in the lymphoid tissues between animals infected with the same virus strain (Fig 6B–6E). We then stained histological sections of the tissues infiltrated with tumor cells for BZLF1 and gp350. All infected tissues contained cells expressing both the early and the late marker of lytic replication but the ratio between EBER and BZLF1 or EBER and gp350 proved to be globally higher in the mice infected with the virus devoid of the BART miRNAs. However, the differences in BZLF1 expression between the two viruses, that proved to be statistically significant, were more pronounced than for gp350 that indeed failed to reach statistical significance (Fig 6D and 6E). One possible explanation for this result is that dead gp350-positive cells are rapidly eliminated *in vivo* but not in *vitro*. We also attempted to generate LCLs with the serum from euthanized animals. However, we could generate only one LCL with the serum from a mouse infected with WT virus that did not display the highest viral titers. We conclude that the viral DNA measured in the serum does not derive from infectious virions, but rather probably from decayed infected cells. This observation is concordant with the fact that free virions are captured by B cells [21]. We also stained the tissues for LMP1 and EBNA2 expression and found that, although immunohistochemistry is not an entirely reliable quantitative assay, mice infected with ΔAll express LMP1 at much higher levels than mice infected with wild type viruses (Fig 6B). However, the percentage of infected cells that expressed LMP1 or EBNA2 among the EBV-infected population showed no difference between wild type- and ΔAll-infected mice (Fig 6F and 6G). We then attempted to confirm these observations in another set of non-humanized immuno-compromised mice. The rationale behind this experiment was to evaluate cell growth in the absence of a functional immune system that might reduce cell growth, particularly in mice infected with the ΔAll mutant that produces more lytic antigens. To this end, we injected i.p. two sets of independent peripheral B cells exposed to wild type or ΔAll viruses. In that case, we terminated the experiment at week 5 to exclude tumor overgrowth and allow more direct comparison between tumor burdens. We found that 7 out of 7 animals infected with ΔAll developed macroscopically visible tumors, mainly in the pancreas and to a lesser extent in the kidney and liver, compared to 3 out of 7 in animals infected with wild type virus (S6A Fig and S1 Table). Importantly, the tumor burden was much higher in mice infected with the ΔAll knockout and these differences were statistically significant (S6B Fig). Whilst the tumor burden concentrated in the spleen in humanized mice, this organ was spared in the non-humanized counterparts, as was expected in the absence of human hematopoietic transplantation. There was no difference in the incidence of tumors between the mice groups infected with the 2 different B cell samples. As previously observed, the tumor cells expressed EBER, EBNA2, LMP1, BZLF1 and gp350 (S6C–S6H Fig). Here again there was no statistical difference in the density of EBER-positive cells within the tumors. In the same vein, the percentage of EBNA2-positive cells was similar in either type of virus infection and lytic replication was clearly stronger in mice infected with M81/ΔAll. There was, however, a difference in the proportion of EBER-positive cells expressing LMP1, the latter being higher in mice infected with M81/ΔAll, suggesting that these cells were selected against in immunocompetent mice. As was the case in humanized mice, the proportion of strongly LMP1-positive cells was much higher in mice infected with M81/ΔAll (S6C Fig). In summary, animals infected with a virus that lacks the BART miRNAs showed increased spontaneous lytic replication and frequently accelerated tumor formation *in vivo*, accompanied by increased LMP1 production.

**Table 1:** Macroscopic and histological features in EBV-infected humanized mice.

Length of infection (in weeks)	Mouse tag	Spleen weight (mg)	EBV+ tumors			Degree of EBV + lymphoid infiltration in organs		
			Spleen	Other organs		Liver	Kidney	Pancreas
			Size of the cut section (mm)	Location	Size of the cut section (mm)			
6	M81-1	140	-	-	-	-	-	-
8	M81-2	109.3	3X4	Pancreas	3X3	+ +	-	+ + +
8	M81-3	430.2	7X5,4X3,3X3	-	-	+ + +	-	-
8	M81-4	112	3X3	-	-	+ +	+ +	-
8	M81-5	183.8	-	-	-	+ +	-	-
6	M81ΔAll-1	285.3	3X4	-	-	+ +	+ +	-
6	M81/ΔAll-2	306.4	3x3, 3x3	-	-	+ +	+ + +	+ + +
6	M81/ΔAll-3	428	6X5,5X5	-	-	+ + +	-	-
6	M81/ΔAll-4	155	2X3;2X1	-	-	+ + +	-	+ +
8	M81/ΔAll-5	51.6		Pancreas	3x2	+ +	-	+ + +
8	M81/ΔAll-6	234.4	2X2,4X3, 4X4	-	-	+ +	+ + +	-
8	M81/ΔAll-7	87	-	Pancreas	5X5	+	-	+ + +

Evaluation area is 0.04μm^2^ corresponding to Fig 6B

+: Less than 10 EBV-infected cells per high power microscopic field

++: Between 10 and 100 infected cells per high power microscopic field

+++: More than 100 infected cells per high power microscopic field

### B cells infected with a virus devoid of BART miRNAs display a complex apoptotic status and modulate the expression of pro- and anti-apoptotic molecules

**Fig 7:**
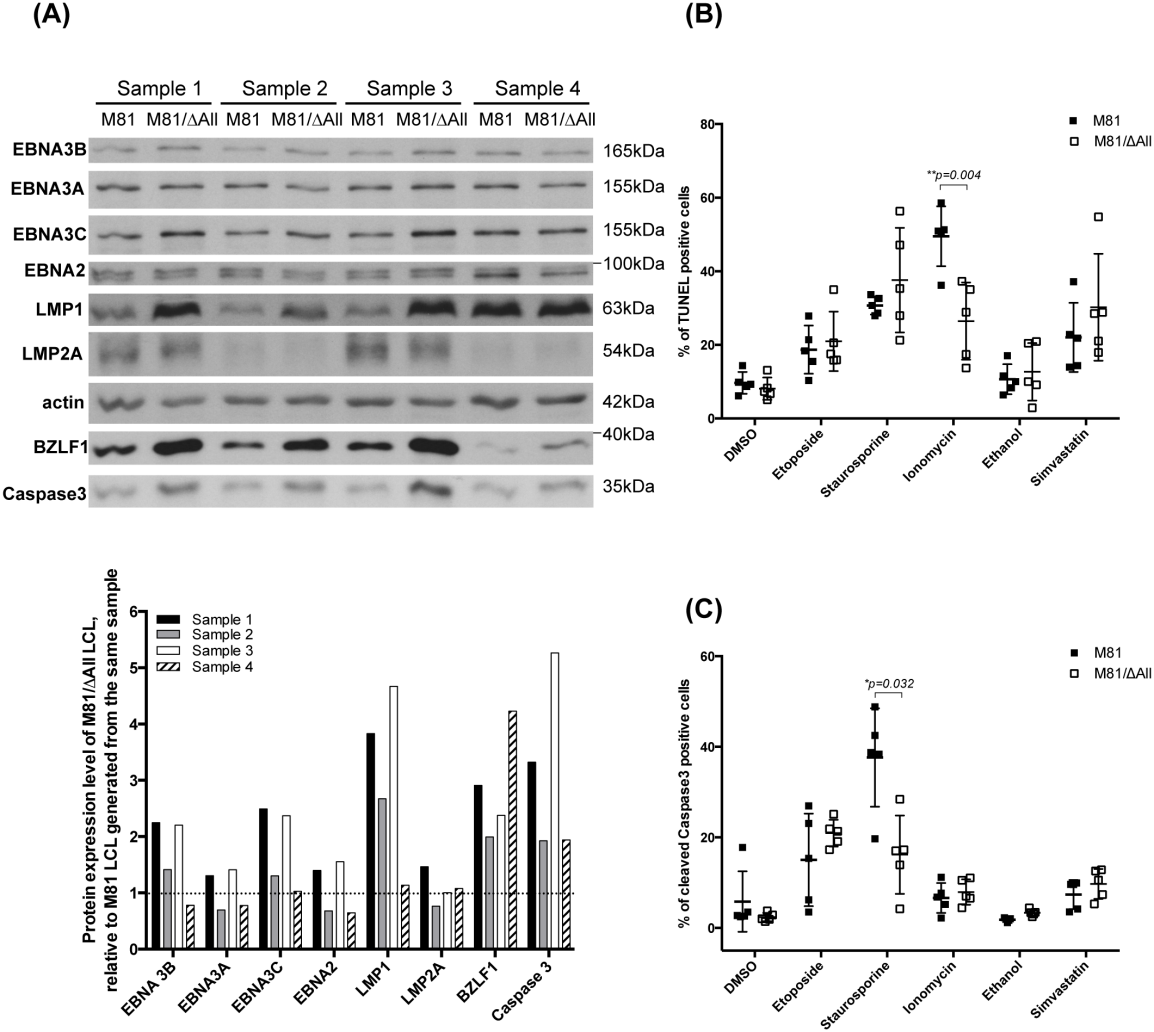
B cells infected by viruses that lack the BART miRNAs express higher levels of caspase 3 and LMP1 and are more resistant to drugs that induce mitochondria-mediated apoptosis. (A) LCLs transformed by M81 or M81/ΔAll from 4 independent donors were subjected to immunoblotting with antibodies specific to viral latent proteins (EBNA3A, 3B, 3C, EBNA2, LMP1 and LMP2A), viral lytic proteins (BZLF1), caspase 3, and actin. The levels of expression of these proteins are also represented in a bar chart. (B and C) Apoptosis was induced in five pairs of LCLs transformed by M81 or M81/ΔAll. Scatter plots represent the percentage of apoptotic cells as determined by TUNEL assays (B) or by immunostaining with antibodies directed against cleaved caspase 3 (C). DMSO or ethanol-treated samples were used as controls. P values lower than 0.05 obtained after paired t-student tests are indicated. Please also refer to S7 and S8 Figs.

The accelerated tumor growth in animals infected with the ΔAll mutant could be explained by a shorter doubling time of B cells infected with this virus. Therefore, we assessed the transforming ability of M81/ΔAll at low cell density and low MOI. We infected primary B cells from 5 independent peripheral blood donors and did not find any difference in transforming ability between wild type and mutant viruses (S7 Fig). We also used western blot to quantify expression of latent genes implicated in B cell growth. We found no difference in EBNA2, EBNA3A, or LMP2 expression. However, EBNA3B and EBNA3C were expressed at mildly higher levels in 3 out of 4 LCLs infected with the virus devoid of the BART miRNAs (Fig 7A). Western blots performed with a LMP1-specific antibody showed a clearly increased expression of this protein in 3 out of 4 cell samples infected with ΔAll mutant, relative to wild type controls. We also assessed LMP1 expression in 3 additional LCL triplets that were generated with wild type M81, the ΔAll mutant and the ΔAll revertant and found that LMP1 expression was identical in both wild type and revertant, but strongly increased in the LCL generated with the ΔAll mutant in 2 out of 3 cases, the remaining case showing a minor LMP1 increase in the ΔAll mutant (S8A Fig). It is interesting to note that LMP1 expression varied in LCLs infected with wild type M81 varied markedly between blood samples, with a minority of LCLs showing a much stronger expression. In these latter cases only, the absence of BART did not or only hardly increased LMP1 expression that was already high in the wild type LCL, suggesting that cellular polymorphisms modulate LMP1 expression. Highly variable LMP1 transcription rates in LCLs infected with the same virus were previously reported [27]. We also addressed the relationship between LMP1 expression and lytic replication by infecting 2 additional blood samples with wild type M81, the ΔAll mutant as well as with a mutant that lacks BZLF1 and BRLF1 and is replication-deficient (M81/ΔZR). We confirmed that BZLF1 expression was higher in the LCLs generated with the ΔAll mutant than in the wild type control. As expected the LCLs generated with M81/ΔZR did not replicate at all. In both samples LMP1 expression was identical in the LCLs generated either with wild type virus or with M81/ΔZR and was weaker than in the LCLs infected with ΔAll (S8B Fig). Altogether, we found that LMP1 expression was stronger after infection with ΔAll in 7 out of 9 independent LCLs.

The BART miRNAs were previously found to increase resistance to apoptosis though inhibition of caspase 3 expression [9]. Therefore, we gauged caspase 3 expression at the protein level using a western blot analysis in infected cells from 4 different donors. This assay showed a 2 to 5-fold increase in caspase 3 expression in all cases. We also assessed the ability of LCLs to withstand an apoptotic stress by incubating the cell lines with a panel of apoptosis-inducing drugs including Ionomycin, Staurosporine, Simvastatin and Etoposide [28]. The levels of apoptosis were assessed by TUNEL or caspase 3 cleavage assays. We could not identify significant differences between LCLs infected with the mutants or with the controls after treatment with Etoposide or Simvastatin (Fig 7B and 7C). However, whilst treatment with Ionomycin gave rise to more apoptosis in LCLs infected with wild type viruses in TUNEL assays, staining with caspase 3 revealed increased apoptosis in wild type LCLs treated with Staurosporine.

## Discussion

The study of spontaneous EBV lytic replication has been hampered by the propensity of the virus to enter latency in infected cells. LCLs initiate some degree of lytic replication after treatment with chemicals such as TPA or butyrate [29]. Some non-lymphoid cell lines such as 293 cells can support lytic replication after transfection with BZLF1 [30]. Infection of primary epithelial cells gives rise to spontaneous lytic replication but the efficiency of infection remains low and these cells are difficult to grow in large numbers [31,32]. Thus, tractable experimental systems have not been available for a long time. However, M81, a virus isolated from an NPC patient replicates strongly in primary B cells isolated from any individual tested so far [21]. Furthermore, M81 is amenable to a genetic analysis after its cloning as a bacterial artificial chromosome [21]. We addressed the function of the BART miRNAs by constructing viruses that evince partial or complete deletions of this locus, as well as a revertant thereof. We found that the BART miRNAs negatively regulate spontaneous lytic replication in B cells, as their excision from the M81 viral genome gives rise to an increase in spontaneous lytic replication *in vitro* and *in vivo* in humanized mice. This phenotypic trait disappears in the revertant virus or upon complementation. The BART miRNAs seem to target BZLF1 directly as its mRNA is recruited more efficiently to the RISC in cells infected by wild type virus than in LCLs generated with the BART miRNA knockout virus and expression of a luciferase gene fused to BZLF1 3’UTR is lower in LCLs generated with wild type virus relative to LCLs generated with the ΔAll virus. However, the difficulties to transfect primary LCLs with high efficiency somehow qualifies the latter result. Typically, miRNAs and their cognate targets are expressed in the same cells and the miRNA down-regulate protein expression in all cells that express them. In LCLs, the BART miRNAs are expressed in latently infected cells that do not express the BZLF1. Therefore, the BART miRNAs can only exert their function on BZLF1 in the minority of cells that initiate lytic replication in a given LCL. Thus, they do not directly control lytic replication but come into play only in cells that have already initiated BZLF1 synthesis. Such a scenario fits with the observation that the number of spontaneously replicating cells in LCLs infected with M81/ΔAll does not exceed 15%. The remaining 85% are devoid of BART miRNAs but nevertheless remain BZLF1-negative. However, in cells that have already initiated lytic replication through expression of BZLF1, the expression of the BART miRNAs apparently needs to be lower than in non-replicating cells. This, combined with the observation that individual replicating cells in LCLs infected with wild type or with M81/ΔAll express the BZLF1 protein at the same level suggests that the halved level of BART miRNAs present in replicating cells infected with wild type viruses is too low to efficiently down-regulate BZLF1 protein. This would mean that the expression of the BART miRNAs needs be lower than in latent cells, but does not need necessarily need to be completely extinguished.

It remains unclear at this point whether the BART miRNAs are actively downregulated in replicating cells through an unknown active molecular mechanism, or whether the expression of the BART miRNA is stochastically distributed within the different cells of a LCL, with replication taking place in cells that happen to express low levels of these non-coding small RNAs. We previously observed that B cells infected with M81 sustain lytic replication over long periods of time, exceeding 3 months of continuous cell culture growth [21]. However, even in these cells, lytic replication eventually stopped. It is interesting to note that the BART miRNAs, probably independently of their effects on BZLF1, also interfere with the mechanisms that control the long-term ability of a lymphoid cell to support lytic replication, as LCLs generated with M81/ΔAll keep producing virus when LCLs established from the same patient with wild type EBV do not anymore. However, replication in LCLs generated with M81/ΔAll also decreases with time, suggesting that the BART miRNAs accelerate this long-term mode of control of lytic replication but are independent of it. Thus, the BART miRNAs could interfere with lytic replication in several ways, negatively modulating lytic replication at its onset through its effect on BZLF1, but also influencing the long-term ability of the LCL to support lytic replication by interfering with the selection process that favors latency.

We then turned our attention to the miRNAs within the cluster that affect BZLF1 expression. Because the cluster encodes 22 miRNAs, we addressed the role played by the subcluster 1, subcluster 2 and miR-BART2 in this process. We found that viruses devoid of the subcluster 2 or of miR-BART2 do not differ from wild type viruses in their ability to control the expression of BZLF1. However, a virus that lacks both subcluster 1 and subcluster 2 expressed more BZLF1 than those infected with a subcluster 1 deletion mutant. This demonstrates that subcluster 1 plays a predominant role in the control of lytic replication but also that subcluster 2 contributes to this process. We did not identify any difference between the ΔC1C2 and the ΔAll viruses in terms of BZLF1 protein expression. Thus, miR-BART-2 does not seem to be implicated in the onset of lytic replication although it clearly modulates BALF5 expression as previously shown and confirmed in the presented study [26]. Two BART miRNAs that belong to subcluster 2 have been proposed to control lytic replication through modulation of BZLF1 and BRLF1 expression. Jung et al. demonstrated that miR-BART20-5p suppressed lytic replication through direct targeting of BZLF1 and BRLF1 mRNAs in a variety of EBV-positive epithelial cell lines [18]. MiR-BART20-5p is not or barely expressed in EBV-transformed LCLs and is thus unlikely to play a substantial role in spontaneous lytic replication in B cells [10]. MiR-BART18-5p was found to repress lytic replication in anti-Ig-treated Akata Burkitt’s lymphoma cell lines and in LCLs induced by TPA through its ability to target MAP3K2 [20]. These models display obvious differences with the spontaneous replication of LCLs that could explain the relatively minor role that we ascribed to the subcluster 2. Therefore, it is possible that miR-BART18-5p plays an essential role in induced but not in spontaneous lytic replication. Our data point to a control of BZLF1 expression shared by multiple BART miRNAs whose individual contribution might be very limited and undetectable in viruses lacking a single miRNA. In such a case, only deletion of a subset of BART miRNAs could reveal their effect on BZLF1 expression and lytic replication.

We also evaluated the role played by the BART miRNAs in the control of EBV-mediated transformation. We found that the deletion of the BART cluster does not influence the transformation abilities of the virus *in vitro*. Its impact in humanized mice was more complex to assess. Mice infected with ΔAll or with wild type viruses developed similar tumor burdens. However, this tumor load was already present after 5 weeks of infection in 4 out of 7 mice infected with ΔAll, whilst it took two additional weeks in the remaining animals. Importantly, mice infected with the BART miRNA knockout experienced a higher level of lytic replication that should theoretically allow infection of a larger number of B cells, thereby increasing the ensuing tumor mass. However, higher lytic replication might also boost the immune response against replicating cells. Therefore, we turned to non-humanized immunocompromized mice that cannot mount an immune response and do not have a large reservoir of EBV-negative resting B cells that can be infected by the viruses produced by a replicating B cell. We find that injection of freshly isolated peripheral blood B cells exposed to viruses and injected intraperitoneally gives rise to lymphoid tumors. This experiment allowed direct comparison of the transforming capacities of the virus and of the mutant. It showed that the tumor incidence is more than twice as high in animals treated with B cells infected by ΔAll and that the tumor burden was on average 5 times higher relative to B cells exposed to wild type virus.

We conclude that the absence of BART miRNAs efficiently supported the growth of EBV-transformed cells. This apparently contradicts numerous studies that implicated the BART miRNAs in EBV-induced epithelial carcinogenesis. Here again, the much higher expression levels in EBV-associated carcinomas needs to be considered. Along the same line, a recently established xenograft model in NSG mice that clearly implicates the BART miRNAs in tumorigenesis uses cells that express the BART miRNAs at even higher levels than in NPC [33]. The expression levels of some EBV latent proteins were also moderately increased in some LCLs transformed by the M81/ΔAll mutant. Although this might have contributed to the increased transforming abilities of the mutant, this increase was inconstant and lower in intensity than observed for BZLF1 or LMP1. This suggests that the effect on these latent proteins was indirect. LMP2A was suggested to be a target of the BART miRNAs but we could not confirm this observation in our experimental system [34]. We also evaluated the impact of the BART miRNAs on apoptosis or more generally cell death in infected B cells. We could not observe any increase in apoptosis in LCLs generated with the BART miRNA-negative virus, but to the contrary a moderate protective effect after treatment with some pro-apoptotic drugs such as Staurosporine. Ionomycin induced a higher level of cell death as assessed by an increased number of cells in TUNEL assay but did not modify the level of cleaved caspase 3. Therefore, it is unlikely to reflect an increased level of apoptosis. Reciprocally, Staurosporine increased the percentage of cleaved caspase 3 but not the percentage of positive cells in TUNEL assays, suggesting that the cells entered the apoptosis pathway but could not complete it. We then evaluated the influence of BART miRNAs on some proteins involved in the regulation of apoptosis in EBV-infected cells. We found that the caspase 3 protein is increased after excision of the BART miRNA cluster in LCLs infected with M81/ΔAll. Caspase 3 has been found as a direct target of the BART miRNAs in Burkitt’s lymphoma cells but a more recent study performed on the NPC C666 cell line could not confirm these results [9,17]. This raises the intriguing possibility of a different mode of interaction between the caspase 3 mRNA and the BART miRNAs in different cell lineages. Our own results cannot distinguish between a direct and an indirect effect of the BART miRNAs on caspase 3 expression.

Importantly, the LCLs that showed increased caspase 3 protein production also expressed the LMP1 protein at higher levels than the controls. This confirms previous studies that identify this viral oncoprotein as a direct target of the BART miRNAs [25,35,36]. Importantly, although immunohistochemistry is not an accurate quantitative method, we found that LMP1 is expressed at clearly higher levels in mice infected with ΔAll and this event is likely to have boosted cell growth. LMP1 has been found to facilitate extrinsic apoptosis through its ability to increase the expression of CD95 [37]. Furthermore, the LMP1 transmembrane domain activates apoptosis through activation of the unfolded protein response [38]. However, LMP1 can also protect against apoptosis through induction of BCL2A1, a member of the BCL2 family of proteins [38], or though inactivation of the p53 protein [39]. As EBV-infected cells express LMP1, the anti-apoptotic effect of this viral protein seems to predominate in infected cells [38]. In LCLs that carry the M81/ΔAll mutant, the increase in LMP1 might predominate over the induction of pro-apoptotic proteins caused by the absence of BART miRNAs. Interestingly, the BART miRNAs were found to have anti-apoptotic properties in Burkitt’s lymphoma cells in which apoptosis was induced by the loss of the EBV genome with the help of a dominant negative version of EBNA1. In that case, LMP1 was not present in cells transfected with the BART miRNAs. It is interesting to note that LMP1 is rarely expressed in EBV-associated carcinomas and this might reflect the repressive effects of the BART miRNAs that are produced at much higher levels in this context [2,5]. The BART cluster offers a good example of how the expression level influences the functions of miRNAs. They also show that miRNAs can have a marked influence on cellular functions even if expressed at seemingly low levels. However, it is important to note that the BART miRNAs are expressed on average at slightly higher levels than the BHRF1 miRNAs in LCLs or even than some crucial cellular miRNAs such as those belonging to the let7 family in hematopoietic stem cells in which miRNAs play a crucial role in cell differentiation [10,40].

In conclusion, we used recombinant viruses to reveal functions of the BART miRNA locus that result from multiple, sometimes even contradictory alterations of viral and cellular functions in cells infected *in vitro* and *in vivo*. This obviously reflects their high number, but also the fact that they collaborate to downregulate targets such as BZLF1 as shown in the present paper, or NDGR1 as previously reported [41]. Virus knockouts that lack single BART miRNA or a subset of them will provide useful tools to dissect their multiple and intricate molecular functions.

## Materials and Methods

### Ethics statement

All human primary B cells used in this study were isolated from anonymous buffy-coats purchased from the Blood Bank of the University of Heidelberg. No ethical approval is required. All animal experiments were performed in strict accordance with German animal protection law (TierSchG) and were approved by the federal veterinary office at the Regierungspräsidium Karlsruhe, Germany (Approval number G156-12). The mice were housed in the class II containment laboratories of the German Cancer Research and handled in accordance with good animal practice with the aim of minimizing animal suffering and reducing mice usage as defined by Federation of European Laboratory Animal Science Associations (FELASA) and the Society for Laboratory Animal Science (GV-SOLAS).

### Cell lines and primary cells

HEK293 cell line is a neuro-endocrine cell line obtained by transformation of embryonic epithelial kidney cells with adenovirus (ATCC: CRL-1573) [42,43]. DG-75 is an EBV-negative cell line that was established from a pleural effusion of a patient with a primary abdominal lymphoma that resembled Burkitt’s lymphoma (ATCC: CRL-2625) [44]. Peripheral blood mononuclear cells from buffy coats purchased from the blood bank in Heidelberg were purified on a Ficoll cushion and CD19-positive primary B-lymphocytes were isolated using M-450 CD19 (Pan B) Dynabeads (Dynal) and were detached using Detachabead (Dynal). WI38 are primary human lung embryonic fibroblasts (ATCC: CCL-75). All cells were routinely cultured in RPMI-1640 medium (Invitrogen) supplemented with 10% fetal bovine serum (FBS)(Biochrom), and primary B cells were supplemented with 20% FBS until establishment of LCLs.

### Oligonucleotides

All synthesized oligonucleotides used for cloning or PCR are listed in S2 Table.

### Construction of recombinant viruses and virus production

The wild type EBV strain M81 is available as a recombinant BACMID [21]. The viral genome was cloned onto a prokaryotic F-plasmid that carries the chloramphenicol (Cam) resistance gene, the gene for green fluorescent protein (GFP), and the Hygromycin resistance gene (B240). All PCR primers used for PCR cloning or chromosomal building are listed in S2 Table and are based on the M81 EBV sequence (GenBank accession number KF373730.1). Deletion of the miR-BART subcluster 1 (deletion from nt 139133 to nt 140132) generated ΔC1; deletion of the miR-BART subcluster 2 (deletion from nt 145492 to nt 148777) gave rise to ΔC2. These mutations were achieved by homologous recombination of the recombinant virus with a linear DNA fragment that encodes the kanamycin resistance gene, flanked by Flp recombination sites, and short DNA regions homologous to the regions immediately outside of the deletion to be obtained, as described [45]. The double knockout BART miRNA subcluster 1 plus subcluster 2 (ΔC1C2) was obtained by excising the kanamycin cassette present in ΔC1 with FLP recombinase, followed by a deletion of BART miRNA subcluster 2 via linear targeting with the PCR product that yielded ΔC2. The ΔAll mutant that lacks all BART miRNAs was obtained by exchanging the miR-BART-2’s seed region with an unrelated sequence in the ΔC1C2 recombinant virus using chromosomal building [45]. This mutagenesis generated an additional AclI restriction site and digestion with this enzyme allows distinction between the mutant and the wild-type sequences (S1 Fig). We applied the same strategy to the wild type M81 BAC to obtain a recombinant EBV that lacks mir-BART-2 only (Δb2). We constructed a revertant of ΔAll using chromosomal building on the basis of the original parental M81/ΔAll Bacmid before passaging in 293. Here the complete BART miRNA locus, from the miR-BART subcluster 1 to miR-BART2, was cloned from the M81 BAC and reintroduced into the M81/ΔAll BAC genome.

We introduced the rat CD2 gene under the control of an EA-D promoter into the BXLF1 gene of the M81 genome (nt 131044 to nt 133362) by homologous recombination using a linear vector that included the kanamycin resistance cassette as a selection marker. The disruption of BXLF1 gene does not interfere with the growth of LCLs [46,47]. Upon induction of the lytic replication, CD2 is expressed at the surface of replicating cells. CD2-positive cells can be pulled down with a specific monoclonal antibody (OX34) coupled with anti-mouse IgG Dynabeads and submitted to protein or RNA extraction.

### Stable transfection of EBV-BAC and plasmid rescue into *E*. *coli*

Recombinant EBV plasmids were lipotransfected into HEK293 cells using Metafectene (Metafectene, Biontex) and the selection of stable 293 cell clones carrying the recombinant EBV plasmid was achieved by adding hygromycin to the culture medium (100 μg/ml) as previously described [48]. To assess the genome integrity of recombinant EBV within the stable clones, the circular EBV genomes present in these cells were extracted using a denaturation-renaturation method [49] and transferred into the *E*.*Coli* strain DH10B by electroporation (1000V, 25μF, 200 Ohms). The transformed *E*.*Coli* clones were further assessed by restriction enzyme analysis of plasmid minipreparations.

### Virus induction

293 cells stably transfected with recombinant EBV-BACs were transfected with expression plasmids encoding BZLF1 (p509) and BALF4 (pRA) using the liposome-based transfectant Metafectene (Biontex). Three days after transfection, virus supernatants were collected and filtered through a 0.4 μm filter.

### Complementation experiments

B1124 is a plasmid that contains a bi-directional tetracycline-inducible CMV promoter that encodes a truncated nerve growth factor receptor (NGFR) on one site, and the BART miRNA subcluster 1 and 2 without the PstI repeats and the LF3 gene on the other. B1034 contains only NGFR and was used as a negative control. M81/ΔAll LCLs were electroporated with either B1034 or B1124 and cultured with 1 μg/ml doxycycline for 30 days. NGFR-positive cells were isolated with specific antibodies and used for protein and RNA analyses.

### Quantification of viral titers

To evaluate EBV genome equivalents per milliliter of supernatant, viral supernatants were treated with DNase I. Following a subsequent treatment with proteinase K, we used quantitative real-time PCR analysis (qPCR) with primers and probe specific for the non-repetitive EBV BALF5 gene sequence to measure the EBV copy numbers in the supernatants [50]. The quantification of EBV DNA genome copies per milliliter in the blood of infected mice was performed from genomic DNA extracted from total blood by using regular phenol extraction.

### Real-time RT-PCR

RNA extracted with Trizol from LCLs was reverse transcribed with AMV-reverse transcriptase (Roche) using a mix of random primers The primers and probes used to detect BZLF1 are listed in the S1 table. The PCR and data analysis was carried out using the universal thermal cycling protocol on an ABI STEP ONE PLUS Sequence Detection System (Applied Biosystems). All samples were run in duplicates, together with primers specific to the human GAPDH gene to normalize for variations in cDNA recovery.

BART miRNAs extracted from cells with Trizol were reverse transcribed using specific stem-loop primers and TaqMan miRNA reverse Transcription Kit (Applied Biosystems), as described elsewhere [8]. The sequences of the stem-loop primers, primers and probes are listed in S2 Table. Reverse transcription and amplification of the cellular snoRNA RNU48 was performed in parallel to normalize for cDNA recovery (Assay ID 001006; Applied Biosystems). Real-time PCR was performed on an ABI STEP ONE PLUS Sequence Detection System (Applied Biosystems).

### Virus infections

B cells purified from peripheral blood were exposed to viral supernatant for two hours, then washed once with PBS and cultured with RPMI supplemented with 20% FBS in the absence of immunosuppressive drugs. For transformation assays, the percentages of EBNA2 positive cells in the infected samples were evaluated by immunostaining with a specific antibody at 3 days post-infection (dpi). Cell populations containing 3 or 30 EBNA2-positive cells per well were seeded into 48 wells of U-bottomed 96-well plates that contained 10^3^ gamma-irradiated WI38 feeder cells. Non-infected B cells were used as a negative control. Outgrowth of lymphoblastoid cell clones (LCLs) was monitored at 33 dpi. We also incubated 10^5^ primary B cells with 25ml of cell-free LCL culture supernatants for 2 hours. These cells were plated on 96 well cluster plates at a concentration of 10^3^ cells per well, together with the same number of gamma-irradiated feeder cells.

### Antibodies

We stained infected cells with mouse monoclonal antibodies against BZLF1 (Clone BZ.1), gp350/220 (Clone 72A1), EBNA2 (Clone PE2) and a Cy-3-conjugated goat-anti-mouse secondary antibody (Dianova, Invitrogen). We performed western blots with mouse monoclonal antibodies against BZLF1 (Clone BZ.1), gp350 (Clone OT6), DICER (clone F10, Santa Cruz Biotechnology), LMP1 (clone CS1-4), EBNA2 (clone PE2), and Actin (clone ACTN05,C4, Dianova). We also used rabbit polyclonal antibodies against caspase-3 (Cell Signalling Technology) and rat monoclonal antibodies (kindly provided by Dr. E. Kremmer Helmholtz Zentrum Munich and Dr. F. Grässer, University of Homburg) against Ago2 (clone 11A9;), EBNA3A (clone E3AN-4A5), EBNA3B (clone 6C9), EBNA3C (clone A10), LMP2A (clone 4E11), BALF5 (clone 4C12). Mouse monoclonal antibodies specific to LMP1 (clone S12, BD Pharmingen), BZLF1 (Clone BZ.1) and gp350 (Clone OT6) were used for immunohistochemical staining against EBV proteins in infected murine tissues.

### Immunostaining

Cells were fixed with 4% paraformaldehyde in PBS for 20 min at room temperature and permeabilized in PBS 0.5% Triton X-100 for 2 min except for samples stained for viral glycoproteins (gp350). Cells were incubated with the first antibody for 30 min, washed in PBS three times, and incubated with a secondary antibody conjugated to Cy-3 for 30 min before embedding in 90% glycerol.

### Western blot analysis

Proteins were extracted with a standard lysis buffer (150 mM NaCl, 0.5% NP-40, 1% Sodium deoxycholat, 0.1% SDS, 5 mM EDTA, 20 mM Tris-HCl pH7.5, proteinase inhibitor cocktail (Roche)) for 15 min on ice followed by sonication to shear the genomic DNA. Up to 20μg of proteins denatured in Laemmli buffer for 5 min at 95 degree were separated on SDS-polyacrylamide gels and electroblotted onto a nitrocellulose membrane (Hybond C, Amersham). Proteins extracted to assess gp350 expression were prepared in Laemmli buffer without 2-mercaptoethanol. After pre-incubation of the blot in 3% milk dry powder in PBST (PBS with 0.2% Triton-X100), the antibody against the target protein was added and incubated at room temperature for 1 hr. After extensive washings in PBST, the blot was incubated for 1 hr with suitable secondary antibodies coupled to horseradish peroxidase (goat anti-mouse (Promega), goat anti-rabbit (Life technologies), or rabbit anti-goat (Santa Cruz) IgG). Bound antibodies were revealed using the ECL detection reagent (Pierce).

### RISC immunoprecipitation assay (RISC-IP)

6 × 10^8^ cells were washed twice in ice-cold PBS and subsequently lysed in 5 ml lysis buffer containing 25 mM Tris HCl (pH 7.5), 150 mM KCl, 2 mM EDTA, 0.5% NP-40, 0.5 mM DTT, 200 u/ml RNAse inhibitor and protease inhibitor cocktail (Roche). Lysates were incubated for 30 min on ice and clarified by centrifugation at 20,000 g for 30 min at 4°C. To estimate the recovery of miRNAs after RISC-IP, total RNA was prepared from 10% of the cell lysates using the TRIzol RNA Isolation Reagents (Life technologies) following the manufacturer's instructions. 6 μg of purified Rat-monoclonal anti-hAgo2 antibody (11A9; Helmholtz Zentrum Munich) or of monoclonal anti-BrdU-antibody (Abcam) was mixed with 20 μl of Dynabeads Protein G (Dynabeads Protein G Immunoprecipitation Kit, Life technologies) and subsequently incubated with 2.5 ml of cell lysates for 4–6 hours under constant rotation at 4°C. The beads were then washed four times with IP wash buffer (300 mM NaCl, 50 mM Tris HCl pH 7.5, 5 mM MgCl_2_, 0.1% NP-40, 1 mM NaF) and once with PBS to remove residual detergents. The beads were resuspended with 300μl of proteinase K buffer (100 mM Tris-HCl PH 7,4 / 50 mM NaCl /10 mM EDTA) in the presence of proteinase K (0.33 mg/ml) and incubated for exactly 30 min at 37°C with shaking at 600 rpm and immediately transferred onto ice. Total RNA present in complexes after RISC-IP was purified by using TRIzol RNA Isolation Reagents and dissolved in 50 μl RNase-free water.

### Luciferase reporter assays

The 3’UTR regions of the M81 BZLF1 and BALF5 genes were PCR amplified with the pair of primers listed in S2 table. The PCR products were digested with EcoRI and XhoI and ligated into the firefly luciferase expressing vector, pGL4.5 (Promega), which had firstly been modified to insert EcoRI and XhoI cutting sites behind the luciferase coding region. The luciferase reporter assays were performed by electroporation of 10 million LCL cells with 5μg of a pcDNA3.1-CD2 plasmid that encodes a truncated rat CD2 protein and 10μg of the different luciferase expression plasmids. 48 hours post electroporation, cells were washed twice with PBS and the luciferase activity was measured by the Beetle-Juice firefly luciferase assay system (PJK). The transfected cells were also immunostained with an antibody specific to rat CD2 to evaluate the electroporation efficiency.

### SYBR green real-time PCR

Total RNA isolated from LCLs or equal volumes of RNA post RISC-IP from different samples were reverse transcribed with AMV-reverse transcriptase (Roche) using a mix of random hexamers. The mRNAs of interest were quantified with the Power SYBR green PCR Mix (Life technologies) using primer pairs specific to the gene of interest. Data analysis was carried out with the universal thermal cycling protocol of the ABI STEP ONE PLUS Sequence Detection System (Applied Biosystems).

### Human immune system component reconstitution in mice (huNSG-A2 mice) for EBV infection

We generated humanized mice by intrahepatical injection of human CD34-positive hematopoietic progenitor cells (HPCs) in irradiated (1 Gy) newborn NSG-A2 mice (NOD.Cg-Prkdc^scid^Il2rg^tm1Wjl^Tg (HLA-A2.1) 1Enge/SzJ) (huNSG-A2 mice). CD34-positive HPCs were isolated from human fetal liver tissue (Advanced Bioscience Resources, Alameda, CA, USA) using a human CD34 purifying Microbead kit (Miltenyi Biotec). We used 2 liver samples to generate the 12 humanized mice used in this study. The percentage of human CD45 positive cells was evaluated 12 weeks after HPC transplantation and only mice with more than 40% of human CD45-positive cells were infected. We performed a titration of viral stocks by infecting primary B cells with increasing dilutions. The infected cells were stained at 3 dpi to determine an EBNA2-positive B cells/ml virus titer. In all experiments, we injected intra-peritoneally in each mouse enough viruses to generate 5x10^6^ EBNA2-positive cells as described [21]. Peripheral blood samples were drawn 5 weeks post-infection. Mice were euthanized at week 8 except if signs of animal suffering became apparent and we examined their blood and tissues for signs of viral infection.

### Transformation experiments in non-humanized NSG mice

We isolated human CD19+ B cells from buffy coats and exposed to virus supernatants at a moi sufficient to generate 20% of EBNA2-positive cells for 2 hours at room temperature under constant agitation. The infected cells were collected by centrifugation and washed twice with PBS for two times. 2*10ˆ6 infected primary B cells, equivalent to 4*10ˆ5 infected cells were injected intraperitoneally into NSG mice. The mice were euthanized at 5 weeks post injection, autopsied and their organs were subjected to macroscopic and microscopic investigation.

### Immunohistochemistry

The organs from the studied mice were fixed in 10% formalin overnight and embedded in paraffin blocks. 3-μm-thin continuous sections were prepared and submitted to antigen retrieval at 98°C for 40 min in a 10 mM sodium citrate, 0.05% Tween20 pH 6.0 solution. Bound antibodies were visualized with the Envision+ Dual link system-HRP (Dako). In parallel, adjacent sections were stained with hematoxylin and eosin (H&E). The presence of EBV was detected by *in situ* hybridization with an EBER-specific PNA probe, in conjunction with a PNA detection kit (Dako). Pictures were taken with a camera attached to a light microscope (Axioplan, Zeiss).

### Apoptosis assay

We induced apoptosis in LCLs (5*10^5^ cell per well of a 48-well-plate) transformed by ΔAll or wild type viruses at 40–60 dpi by adding Etoposide (4μg/ml, Sigma Aldrich) or Staurosporine (4μg/ml, Sigma-Aldrich) to the culture medium for 20 hrs. Cells were also treated with Ionomycin (4μg/ml, Sigma Aldrich) for 48 hrs or Simvastatin (2μM, Calbiochem) for 5 days. We included DMSO-treated cells and ethanol-treated cells as controls. Cells were then washed twice with ice-cold PBS, dried on glass slides and fixed with 4% paraformaldehyde in PBS to perform a TUNEL assay that labels apoptotic cells with DNA breaks (Cell Death Detection Kit, TMR red, Roche) following the instruction of manufacturer. Cells were also stained with a rabbit antibody specific for cleaved caspase 3 (Cell signal technology).

### Statistical analysis

All results obtained in *in vitro* studies with LCLs generated by EBV wild type or mutants with B cells from the same blood donors were paired and analyzed by paired student t-test. Unpaired student t-test was applied for analyzing the grouped humanized or non-humanized NSG mice infected by either M81 or M81/ΔAll virus. All p-values were analyzed as 2-tailed and the values equal to 0.05 or less were considered significant unless indicated. We used a Chi square test to analyze the tumor incidence in humanized and non-humanized mice. The statistical analyses were performed with the GraphPad Prism 5 software.

## Supporting Information

S1 Fig Construction of the M81 BART miRNA mutants. (A) Schematic map of a segment from the rM81 genome that encompasses the BART miRNA region. The deletion mutants were obtained by replacing the BART miRNA subcluster 1 and 2 with a kanamycin resistance cassette or by mutating miR-BART-2 so as to introduce an Acl1 restriction site. (B) These restriction analyses from DNA Bacmid minipreparations show the restriction pattern of the M81/ΔC1, M81/ΔC2, M81/Δb2 and M81/ΔAll knockout mutants. The investigated samples include rM81 bacmids after construction in *E*.*coli* or after rescue from the producer cell lines. The viral DNAs were cleaved with HindIII or AclI and separated on an agarose gel. The parental rM81 recombinant EBV plasmid was loaded as a control. The arrows indicate the viral DNA fragments whose sizes differ between the wild type rM81 and the mutants as illustrated in the schematic shown in (A).
(TIF)

S2 Fig BART miRNA and mRNA expression pattern in LCLs infected with the mutated viruses. **(A)** We performed quantitative stem loop RT-PCR with primers specific for multiple BART miRNAs located within the different clusters on LCLs generated with the different mutants. The observed values were normalized to those obtained from LCL transformed by wild type M81 and given in a bars graph. The standard variation of three technical replicates is given. (B) We assessed the expression of BART transcripts in 4 independent LCLs infected with M81 or M81/ΔAll LCLs using quantitative RT-PCR with primers specific for the exon7b of the BART transcript. The recorded values were each normalized relative to those collected from the analysis of the LCL transformed by wild type M81. The boxplot shows the mean value and quartiles of the observed values. A statistical analysis was performed using 2-tailed paired student t test.
(TIF)

S3 Fig LCLs transformed by a virus devoid of the BART miRNAs express BZLF1 at higher levels. (A) We performed immunoblots analyses on extracts from 11 additional LCL pairs transformed with M81 and M81/ΔAll at 40–50 dpi with antibodies specific for BZLF1 and actin (A to K). The relative intensities of the signals was quantified using the ImageJ software and are given as a graph of bars. (B) The boxplot summarizes the BZLF1 protein level in 17 pairs of LCLs transformed by M81 and M81/ΔAll shown in Figs 1, 7 and S3A. We excluded the results of sample G that recorded a 40-fold enhancement of BZLF1 expression in the LCL generated with ΔAll to avoid skewing effects due to an outlier. We used a two-tailed paired student t test to evaluate the statistical significance of the results and show the resulting calculated p value.
(TIF)

S4 Fig Increased viral miRNA expression after RISC immunoprecipitation. (A) Immunoblots with antibodies Ago2 protein performed on RISC immunoprecipitated with an anti-Ago2 antibody. The figure shows the results of a RISC immunoprecipitation performed with LCLs transformed with M81 and M81/ΔAll. (B) The specific RISC immunoprecipitation leads to a massive enrichment in miR-BHRF1-1, as assessed by stem loop qPCR. The results give the difference between the Ct values (ΔCt) obtained after stem loop qPCR performed on the immunoprecipitates obtained with the anti-Ago2 antibody or with the anti-BrdU antibody.
(TIF)

S5 Fig The miRNA subcluster1 is mainly but not exclusively responsible for the control of BZLF1 expression. The figure shows Western blot analyses of one set of LCL generated with one B cell sample and M81, M81/ΔAll, M81/ΔC1, M81/ΔC2, M81/ΔC1C2, M81/Δb2, M81/ΔZR. The proteins were stained with a BZLF1-specific antibody. The LCLs were stained at 42 (A) and 101 (B) days post-infection. The relative intensity of the signals were quantified using the ImageJ software and are also displayed as a graph of bars. One more sample is shown in Fig 5.
(TIF)

S6 Fig B cells infected with EBV deprived of the BART miRNAs accelerate tumor growth after injection into NSG mice. Freshly isolated primary B cells from the peripheral blood were exposed to M81 or M81/ΔAll viruses *in vitro* for 2 hours at room temperature and immediately injected into NSG mice intraperitoneally. B cells from one blood donor infected with wild type or mutant virus were injected into 8 mice, infected B cells from a second blood donor were injected into 6 mice. The experiment was terminated at the end of the 5^th^ week post-injection. The figure shows the tumor incidence in (A) as well as the weight of the main tumor burden that invaded the pancreatic region in (B). (C-H) show the multiple immunohistological stains of the large tumors that developed in the pancreatic area. (C) Continuous tissue sections were stained with hematoxylin and eosin (H&E), immunostained with antibodies specific for BZLF1, gp350, LMP1, EBNA2, or subjected to an *in situ* hybridization with an EBER-specific probe. Two tumors from 2 mice in each group are shown. (D) The number of EBER positive cells per 0.04μm^2^ (surface of the field at high magnification) is given in this boxplot. (E-H) The boxplots display the ratio between (E) BZLF1-, (F) gp350-, (G) LMP1-, or (H) EBNA2-positive cells versus EBER-positive cells.
(TIF)

S7 Fig A BART-miRNAs null mutant retains wild type transformation abilities. We compared the transformation abilities of M81 and M81/ΔAll by counting the number of outgrowing wells in 96 well plates seeded with infected primary B cells containing either 3 or 30 EBNA2-positive cells after 5 weeks. The bar chart shows the arithmetic mean of four independent experiments and their standard deviation.
(TIF)

S8 Fig The enhanced LMP1 protein production in LCLs generated with the M81/ΔAll mutant is independent of lytic replication and disappears after infection with its revertant. (A) The immunoblots shown in Fig 1A were stripped and restained with antibodies specific for LMP1. (B) Extracts from 2 sets of independent LCLs generated with M81, M81/ΔAll or M81/ΔZR viruses were used to perform Western blots with antibodies against LMP1, BZLF1, and actin. The relative intensity of signals of each analysis was quantified using the ImageJ software and are given as a graph of bars.
(TIF)

S1 Table Macroscopic and histological features of NSG mice treated with EBV-infected primary B cells. (DOCX)

S2 Table Oligonucleotides used in this study. (DOCX)

## References

[1] Kieff ED, Rickinson AB (2007) Epstein-Barr Virus and its replication In: Knipe DM HP, Griffin DE, Lamb RA, Martin MA, Roizman B, Straus SE, editor. Field Virology. Philadelphia: Lippincott Williams & Wilkins pp. 2603–2654.

[2] Rickinson AB, Kieff E (2007) Epstein-Barr virus In: Knipe DM, Howley PM, Griffin DE, Lamb RA, Martin MA et al, editors. Fields Virology. 5th Edition ed. Philadelphia: Lippincott Williams & Wilkins pp. 2655–2700.

[3] Pfeffer S, Zavolan M, Grasser FA, Chien M, Russo JJ, et al (2004) Identification of virus-encoded microRNAs. Science 304: 734–736. 15118162 10.1126/science.1096781

[4] Grundhoff A, Sullivan CS, Ganem D (2006) A combined computational and microarray-based approach identifies novel microRNAs encoded by human gamma-herpesviruses. RNA 12: 733–750. 16540699 10.1261/rna.2326106 PMC1440911

[5] Cai X, Schafer A, Lu S, Bilello JP, Desrosiers RC, et al (2006) Epstein-Barr virus microRNAs are evolutionarily conserved and differentially expressed. PLoS Pathog 2: e23 16557291 10.1371/journal.ppat.0020023 PMC1409806

[6] Seto E, Moosmann A, Gromminger S, Walz N, Grundhoff A, et al (2010) Micro RNAs of Epstein-Barr virus promote cell cycle progression and prevent apoptosis of primary human B cells. PLoS Pathog 6: e1001063 10.1371/journal.ppat.1001063 20808852 PMC2924374

[7] Feederle R, Haar J, Bernhardt K, Linnstaedt SD, Bannert H, et al (2011) The members of an Epstein-Barr virus microRNA cluster cooperate to transform B lymphocytes. J Virol 85: 9801–9810. 10.1128/JVI.05100-11 21752900 PMC3196389

[8] Feederle R, Linnstaedt SD, Bannert H, Lips H, Bencun M, et al (2011) A viral microRNA cluster strongly potentiates the transforming properties of a human herpesvirus. PLoS Pathog 7: e1001294 10.1371/journal.ppat.1001294 21379335 PMC3040666

[9] Vereide DT, Seto E, Chiu YF, Hayes M, Tagawa T, et al (2014) Epstein-Barr virus maintains lymphomas via its miRNAs. Oncogene 33: 1258–1264. 10.1038/onc.2013.71 23503461 PMC3690170

[10] Qiu J, Cosmopoulos K, Pegtel M, Hopmans E, Murray P, et al (2011) A novel persistence associated EBV miRNA expression profile is disrupted in neoplasia. PLoS Pathog 7: e1002193 10.1371/journal.ppat.1002193 21901094 PMC3161978

[11] Marquitz AR, Raab-Traub N (2012) The role of miRNAs and EBV BARTs in NPC. Semin Cancer Biol 22: 166–172. 10.1016/j.semcancer.2011.12.001 22178394 PMC3340885

[12] Hsu CY, Yi YH, Chang KP, Chang YS, Chen SJ, et al (2014) The Epstein-Barr virus-encoded microRNA MiR-BART9 promotes tumor metastasis by targeting E-cadherin in nasopharyngeal carcinoma. PLoS Pathog 10: e1003974 10.1371/journal.ppat.1003974 24586173 PMC3937311

[13] Cai LM, Lyu XM, Luo WR, Cui XF, Ye YF, et al (2014) EBV-miR-BART7-3p promotes the EMT and metastasis of nasopharyngeal carcinoma cells by suppressing the tumor suppressor PTEN. Oncogene. 10.1038/onc.2014.341 25347742

[14] Lei T, Yuen KS, Xu R, Tsao SW, Chen H, et al (2013) Targeting of DICE1 tumor suppressor by Epstein-Barr virus-encoded miR-BART3* microRNA in nasopharyngeal carcinoma. Int J Cancer 133: 79–87. 10.1002/ijc.28007 23280823

[15] Choy EY, Siu KL, Kok KH, Lung RW, Tsang CM, et al (2008) An Epstein-Barr virus-encoded microRNA targets PUMA to promote host cell survival. J Exp Med 205: 2551–2560. 10.1084/jem.20072581 18838543 PMC2571930

[16] Marquitz AR, Mathur A, Nam CS, Raab-Traub N (2011) The Epstein-Barr Virus BART microRNAs target the pro-apoptotic protein Bim. Virology 412: 392–400. 10.1016/j.virol.2011.01.028 21333317 PMC3340891

[17] Kang D, Skalsky RL, Cullen BR (2015) EBV BART MicroRNAs Target Multiple Pro-apoptotic Cellular Genes to Promote Epithelial Cell Survival. PLoS Pathog 11: e1004979 10.1371/journal.ppat.1004979 26070070 PMC4466530

[18] Jung YJ, Choi H, Kim H, Lee SK (2014) MicroRNA miR-BART20-5p stabilizes Epstein-Barr virus latency by directly targeting BZLF1 and BRLF1. J Virol 88: 9027–9037. 10.1128/JVI.00721-14 24899173 PMC4136301

[19] Iizasa H, Wulff BE, Alla NR, Maragkakis M, Megraw M, et al (2010) Editing of Epstein-Barr virus-encoded BART6 microRNAs controls their dicer targeting and consequently affects viral latency. J Biol Chem 285: 33358–33370. 10.1074/jbc.M110.138362 20716523 PMC2963350

[20] Qiu J, Thorley-Lawson DA (2014) EBV microRNA BART 18-5p targets MAP3K2 to facilitate persistence in vivo by inhibiting viral replication in B cells. Proc Natl Acad Sci U S A 111: 11157–11162. 10.1073/pnas.1406136111 25012295 PMC4121837

[21] Tsai MH, Raykova A, Klinke O, Bernhardt K, Gartner K, et al (2013) Spontaneous lytic replication and epitheliotropism define an Epstein-Barr virus strain found in carcinomas. Cell Rep 5: 458–470. 10.1016/j.celrep.2013.09.012 24120866

[22] Klinke O, Feederle R, Delecluse HJ (2014) Genetics of Epstein-Barr virus microRNAs. Semin Cancer Biol 26: 52–59. 10.1016/j.semcancer.2014.02.002 24602823

[23] Kuzembayeva M, Chiu YF, Sugden B (2012) Comparing proteomics and RISC immunoprecipitations to identify targets of Epstein-Barr viral miRNAs. PLoS One 7: e47409 10.1371/journal.pone.0047409 23091622 PMC3472983

[24] Dolken L, Malterer G, Erhard F, Kothe S, Friedel CC, et al (2010) Systematic analysis of viral and cellular microRNA targets in cells latently infected with human gamma-herpesviruses by RISC immunoprecipitation assay. Cell Host Microbe 7: 324–334. 10.1016/j.chom.2010.03.008 20413099

[25] Skalsky RL, Corcoran DL, Gottwein E, Frank CL, Kang D, et al (2012) The viral and cellular microRNA targetome in lymphoblastoid cell lines. PLoS Pathog 8: e1002484 10.1371/journal.ppat.1002484 22291592 PMC3266933

[26] Barth S, Pfuhl T, Mamiani A, Ehses C, Roemer K, et al (2008) Epstein-Barr virus-encoded microRNA miR-BART2 down-regulates the viral DNA polymerase BALF5. Nucleic Acids Res 36: 666–675. 18073197 10.1093/nar/gkm1080 PMC2241876

[27] Davies ML, Xu S, Lyons-Weiler J, Rosendorff A, Webber SA, et al (2010) Cellular factors associated with latency and spontaneous Epstein-Barr virus reactivation in B-lymphoblastoid cell lines. Virology 400: 53–67. 10.1016/j.virol.2010.01.002 20153012

[28] Michalak EM, Villunger A, Adams JM, Strasser A (2008) In several cell types tumour suppressor p53 induces apoptosis largely via Puma but Noxa can contribute. Cell Death Differ 15: 1019–1029. 10.1038/cdd.2008.16 18259198 PMC2974267

[29] Jones RJ, Seaman WT, Feng WH, Barlow E, Dickerson S, et al (2007) Roles of lytic viral infection and IL-6 in early versus late passage lymphoblastoid cell lines and EBV-associated lymphoproliferative disease. Int J Cancer 121: 1274–1281. 17520680 10.1002/ijc.22839

[30] Delecluse HJ, Hilsendegen T, Pich D, Zeidler R, Hammerschmidt W (1998) Propagation and recovery of intact, infectious Epstein-Barr virus from prokaryotic to human cells. Proc Natl Acad Sci U S A 95: 8245–8250. 9653172 10.1073/pnas.95.14.8245 PMC20961

[31] Feederle R, Neuhierl B, Bannert H, Geletneky K, Shannon-Lowe C, et al (2007) Epstein-Barr virus B95.8 produced in 293 cells shows marked tropism for differentiated primary epithelial cells and reveals interindividual variation in susceptibility to viral infection. Int J Cancer 121: 588–594. 17417777 10.1002/ijc.22727

[32] Tsang CM, Yip YL, Lo KW, Deng W, To KF, et al (2012) Cyclin D1 overexpression supports stable EBV infection in nasopharyngeal epithelial cells. Proc Natl Acad Sci U S A 109: E3473–3482. 10.1073/pnas.1202637109 23161911 PMC3528537

[33] Qiu J, Smith P, Leahy L, Thorley-Lawson DA (2015) The Epstein-Barr virus encoded BART miRNAs potentiate tumor growth in vivo. PLoS Pathog 11: e1004561 10.1371/journal.ppat.1004561 25590614 PMC4295875

[34] Lung RW, Tong JH, Sung YM, Leung PS, Ng DC, et al (2009) Modulation of LMP2A expression by a newly identified Epstein-Barr virus-encoded microRNA miR-BART22. Neoplasia 11: 1174–1184. 19881953 10.1593/neo.09888 PMC2767219

[35] Riley KJ, Rabinowitz GS, Yario TA, Luna JM, Darnell RB, et al (2012) EBV and human microRNAs co-target oncogenic and apoptotic viral and human genes during latency. EMBO J 31: 2207–2221. 10.1038/emboj.2012.63 22473208 PMC3343464

[36] Lo AK, To KF, Lo KW, Lung RW, Hui JW, et al (2007) Modulation of LMP1 protein expression by EBV-encoded microRNAs. Proc Natl Acad Sci U S A 104: 16164–16169. 17911266 10.1073/pnas.0702896104 PMC2042179

[37] Le Clorennec C, Youlyouz-Marfak I, Adriaenssens E, Coll J, Bornkamm GW, et al (2006) EBV latency III immortalization program sensitizes B cells to induction of CD95-mediated apoptosis via LMP1: role of NF-kappaB, STAT1, and p53. Blood 107: 2070–2078. 16317104 10.1182/blood-2005-05-2053

[38] Pratt ZL, Zhang J, Sugden B (2012) The latent membrane protein 1 (LMP1) oncogene of Epstein-Barr virus can simultaneously induce and inhibit apoptosis in B cells. J Virol 86: 4380–4393. 10.1128/JVI.06966-11 22318153 PMC3318665

[39] Li L, Li W, Xiao L, Xu J, Chen X, et al (2012) Viral oncoprotein LMP1 disrupts p53-induced cell cycle arrest and apoptosis through modulating K63-linked ubiquitination of p53. Cell Cycle 11: 2327–2336. 10.4161/cc.20771 22684299

[40] Bissels U, Wild S, Tomiuk S, Holste A, Hafner M, et al (2009) Absolute quantification of microRNAs by using a universal reference. RNA 15: 2375–2384. 10.1261/rna.1754109 19861428 PMC2779673

[41] Kanda T, Miyata M, Kano M, Kondo S, Yoshizaki T, et al (2015) Clustered MicroRNAs of the Epstein-Barr Virus Cooperatively Downregulate an Epithelial Cell-Specific Metastasis Suppressor. J Virol 89: 2684–2697. 10.1128/JVI.03189-14 25520514 PMC4325718

[42] Graham FL, Smiley J, Russell WC, Nairn R (1977) Characteristics of a human cell line transformed by DNA from human adenovirus type 5. J Gen Virol 36: 59–74. 886304 10.1099/0022-1317-36-1-59

[43] Shaw G, Morse S, Ararat M, Graham FL (2002) Preferential transformation of human neuronal cells by human adenoviruses and the origin of HEK 293 cells. Faseb J 16: 869–871. Epub 2002 Apr 2010. 11967234 10.1096/fj.01-0995fje

[44] Ben-Bassat H, Goldblum N, Mitrani S, Goldblum T, Yoffey JM, et al (1977) Establishment in continuous culture of a new type of lymphocyte from a "Burkitt like" malignant lymphoma (line D.G.-75). Int J Cancer 19: 27–33. 188769 10.1002/ijc.2910190105

[45] Feederle R, Bartlett EJ, Delecluse HJ (2010) Epstein-Barr virus genetics: talking about the BAC generation. Herpesviridae 1(1):6 10.1186/2042-4280-1-6 21429237 PMC3063228

[46] Yoshiyama H, Shimizu N, Takada K (1995) Persistent Epstein-Barr virus infection in a human T-cell line: unique program of latent virus expression. EMBO J 14: 3706–3711. 7641689 10.1002/j.1460-2075.1995.tb00040.x PMC394445

[47] Kanda T, Yajima M, Ahsan N, Tanaka M, Takada K (2004) Production of high-titer Epstein-Barr virus recombinants derived from Akata cells by using a bacterial artificial chromosome system. J Virol 78: 7004–7015. 15194777 10.1128/JVI.78.13.7004-7015.2004 PMC421639

[48] Janz A, Oezel M, Kurzeder C, Mautner J, Pich D, et al (2000) Infectious Epstein-Barr virus lacking major glycoprotein BLLF1 (gp350/220) demonstrates the existence of additional viral ligands. J Virol 74: 10142–10152. 11024143 10.1128/jvi.74.21.10142-10152.2000 PMC102053

[49] Griffin BE, Bjorck E, Bjursell G, Lindahl T (1981) Sequence complexity of circular Epstein-Bar virus DNA in transformed cells. J Virol 40: 11–19. 6270367 10.1128/jvi.40.1.11-19.1981 PMC256591

[50] Feederle R, Neuhierl B, Baldwin G, Bannert H, Hub B, et al (2006) Epstein-Barr virus BNRF1 protein allows efficient transfer from the endosomal compartment to the nucleus of primary B lymphocytes. J Virol 80: 9435–9443. 16973549 10.1128/JVI.00473-06 PMC1617231

