# Supplementary figures and images for: The Epstein-Barr Virus BART miRNA Cluster of the M81 Strain Modulates Multiple Functions in Primary B Cells
Source: PLoS Pathog. 2015 Dec 22;11(12):e1005344. doi: 10.1371/journal.ppat.1005344 (PMC4691206; doi:10.1371/journal.ppat.1005344)

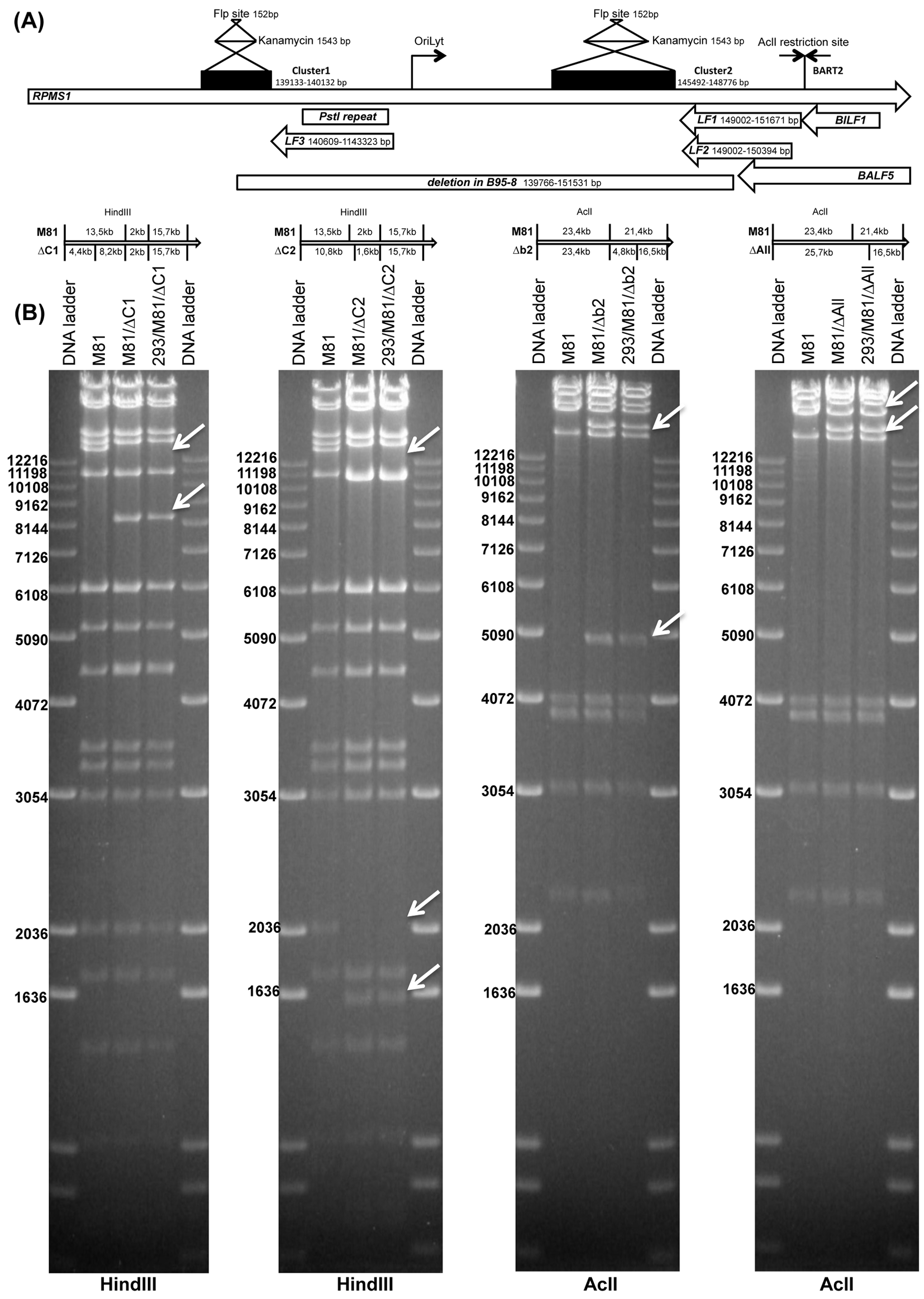

Supplement: S1 Fig — (A) Schematic map of a segment from the rM81 genome that encompasses the BART miRNA region. The deletion mutants were obtained by replacing the BART miRNA subcluster 1 and 2 with a kanamycin resistance cassette or by mutating miR-BART-2 so as to introduce an Acl1 restriction site. (B) These restriction analyses from DNA Bacmid minipreparations show the restriction pattern of the M81/ΔC1, M81/ΔC2, M81/Δb2 and M81/ΔAll knockout mutants. The investigated samples include rM81 bacmids after construction in E.coli or after rescue from the producer cell lines. The viral DNAs were cleaved with HindIII or AclI and separated on an agarose gel. The parental rM81 recombinant EBV plasmid was loaded as a control. The arrows indicate the viral DNA fragments whose sizes differ between the wild type rM81 and the mutants as illustrated in the schematic shown in (A). (TIF) [file ppat.1005344.s001.tif]

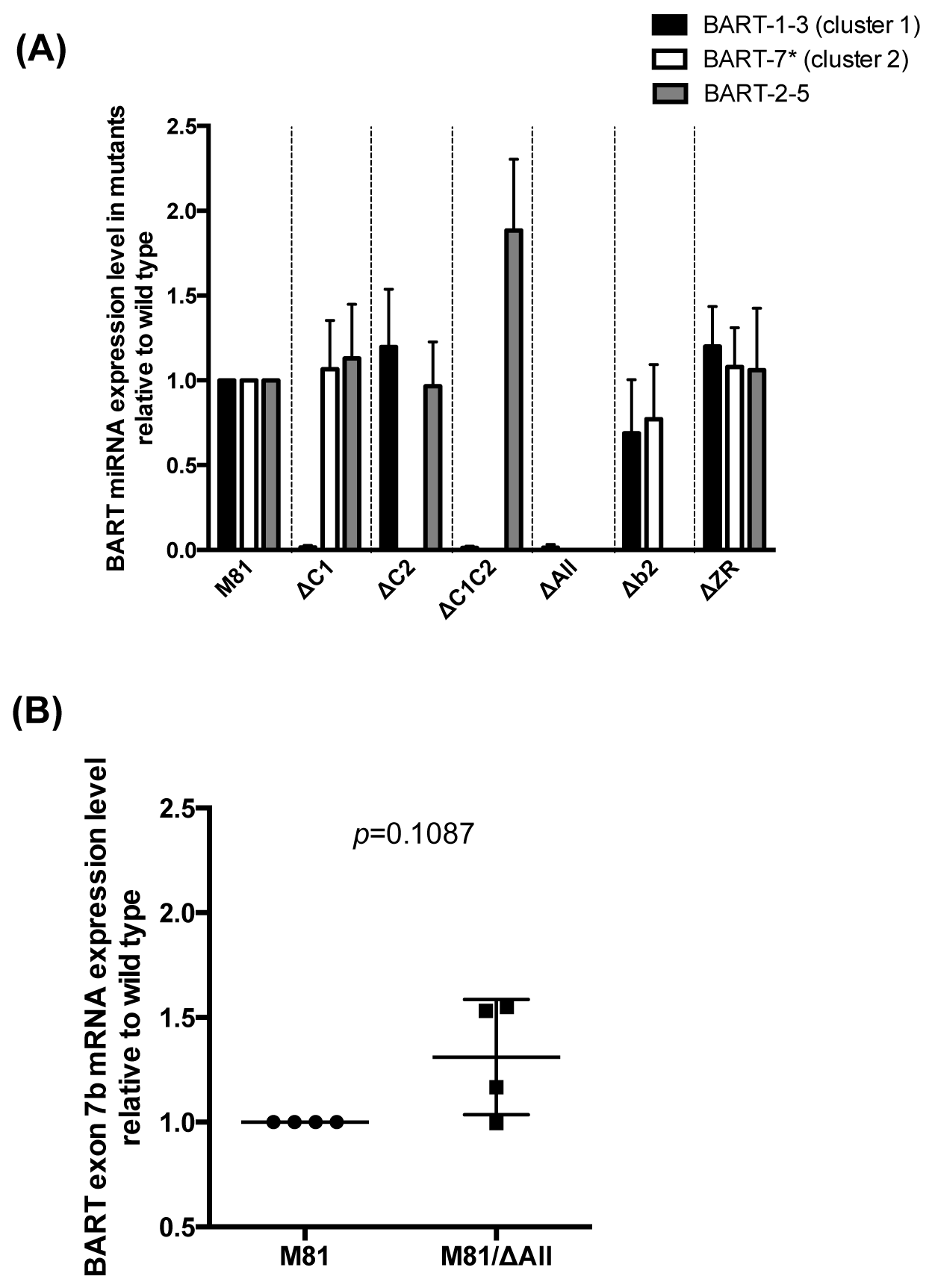

Supplement: S2 Fig — (A) We performed quantitative stem loop RT-PCR with primers specific for multiple BART miRNAs located within the different clusters on LCLs generated with the different mutants. The observed values were normalized to those obtained from LCL transformed by wild type M81 and given in a bars graph. The standard variation of three technical replicates is given. (B) We assessed the expression of BART transcripts in 4 independent LCLs infected with M81 or M81/ΔAll LCLs using quantitative RT-PCR with primers specific for the exon7b of the BART transcript. The recorded values were each normalized relative to those collected from the analysis of the LCL transformed by wild type M81. The boxplot shows the mean value and quartiles of the observed values. A statistical analysis was performed using 2-tailed paired student t test. (TIF) [file ppat.1005344.s002.tif]

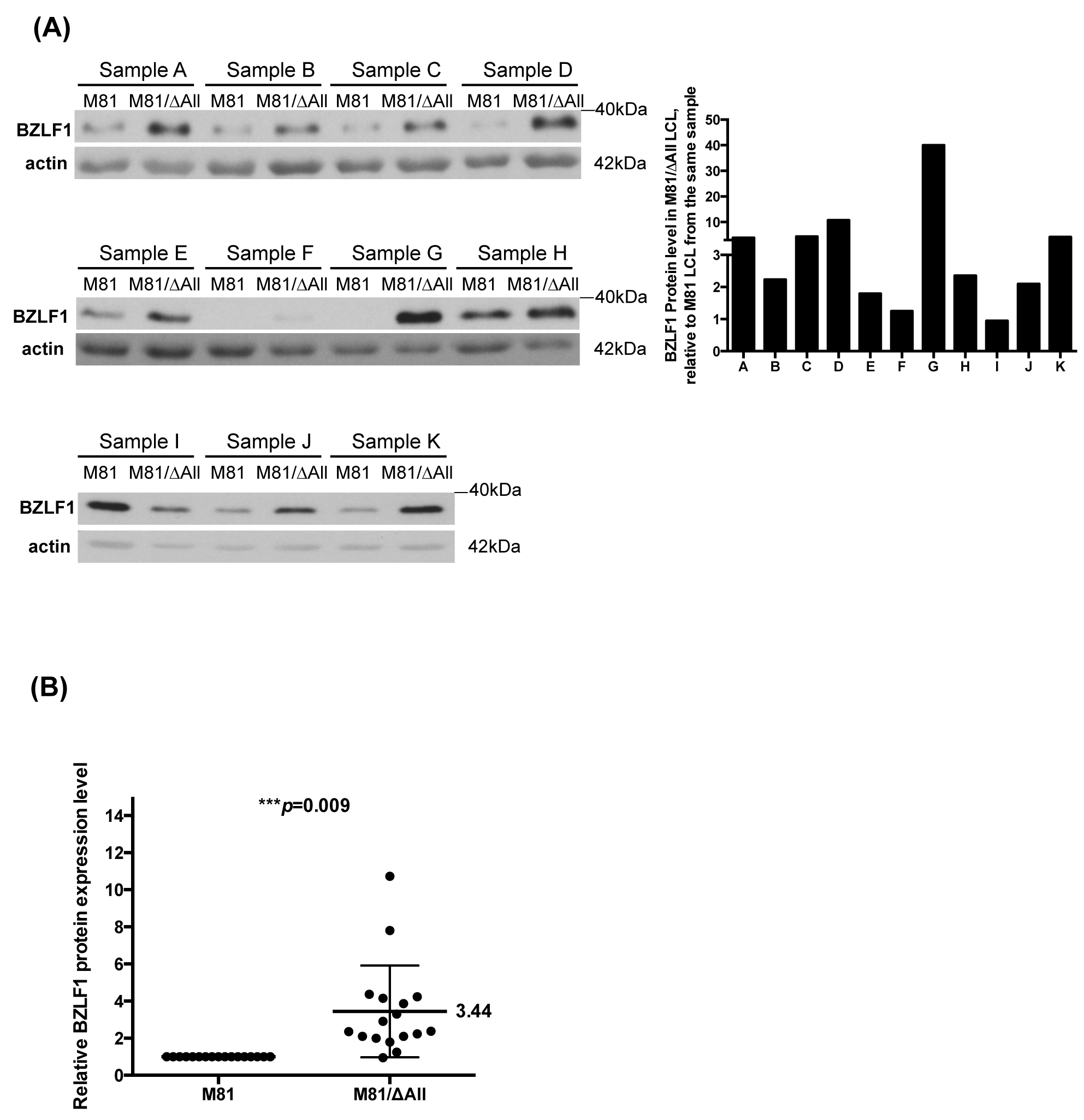

Supplement: S3 Fig — (A) We performed immunoblots analyses on extracts from 11 additional LCL pairs transformed with M81 and M81/ΔAll at 40–50 dpi with antibodies specific for BZLF1 and actin (A to K). The relative intensities of the signals was quantified using the ImageJ software and are given as a graph of bars. (B) The boxplot summarizes the BZLF1 protein level in 17 pairs of LCLs transformed by M81 and M81/ΔAll shown in Figs 1, 7 and S3A. We excluded the results of sample G that recorded a 40-fold enhancement of BZLF1 expression in the LCL generated with ΔAll to avoid skewing effects due to an outlier. We used a two-tailed paired student t test to evaluate the statistical significance of the results and show the resulting calculated p value. (TIF) [file ppat.1005344.s003.tif]

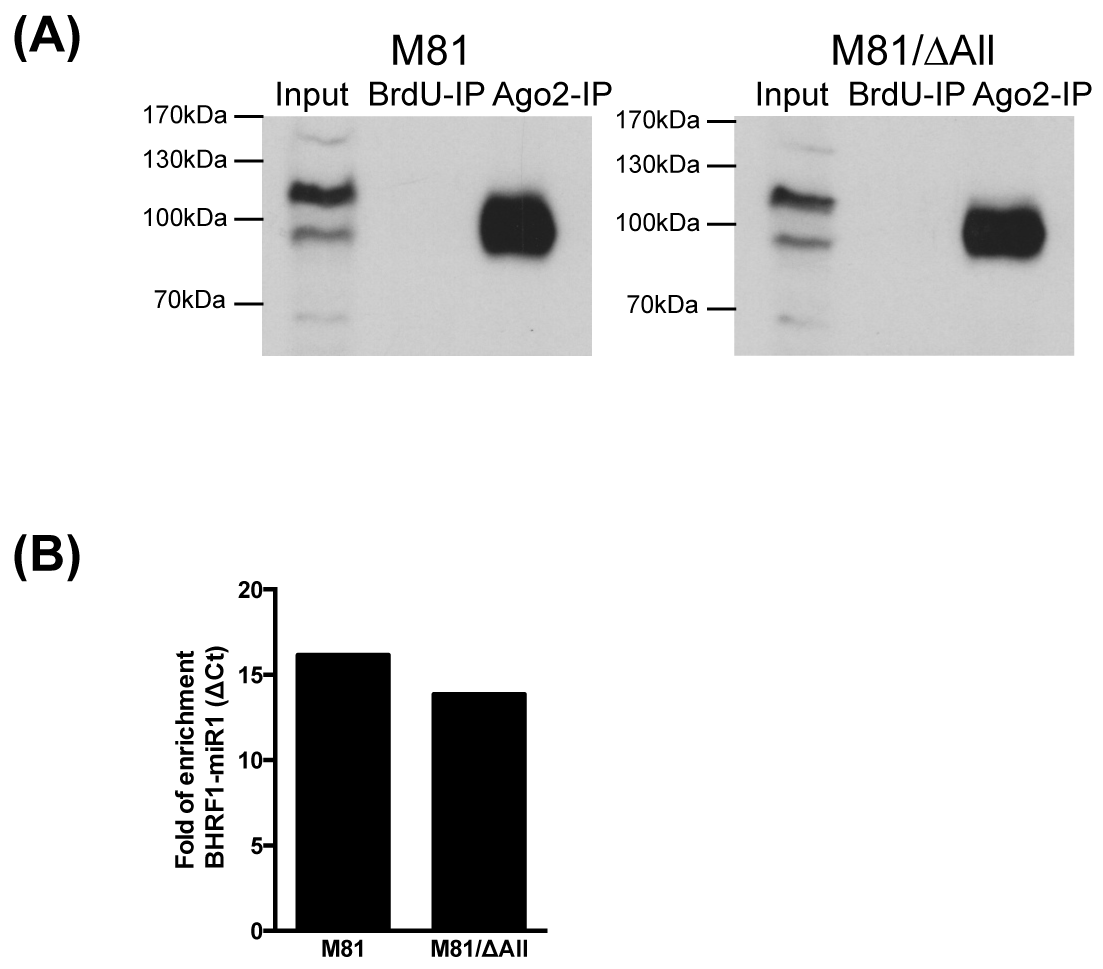

Supplement: S4 Fig — (A) Immunoblots with antibodies Ago2 protein performed on RISC immunoprecipitated with an anti-Ago2 antibody. The figure shows the results of a RISC immunoprecipitation performed with LCLs transformed with M81 and M81/ΔAll. (B) The specific RISC immunoprecipitation leads to a massive enrichment in miR-BHRF1-1, as assessed by stem loop qPCR. The results give the difference between the Ct values (ΔCt) obtained after stem loop qPCR performed on the immunoprecipitates obtained with the anti-Ago2 antibody or with the anti-BrdU antibody. (TIF) [file ppat.1005344.s004.tif]

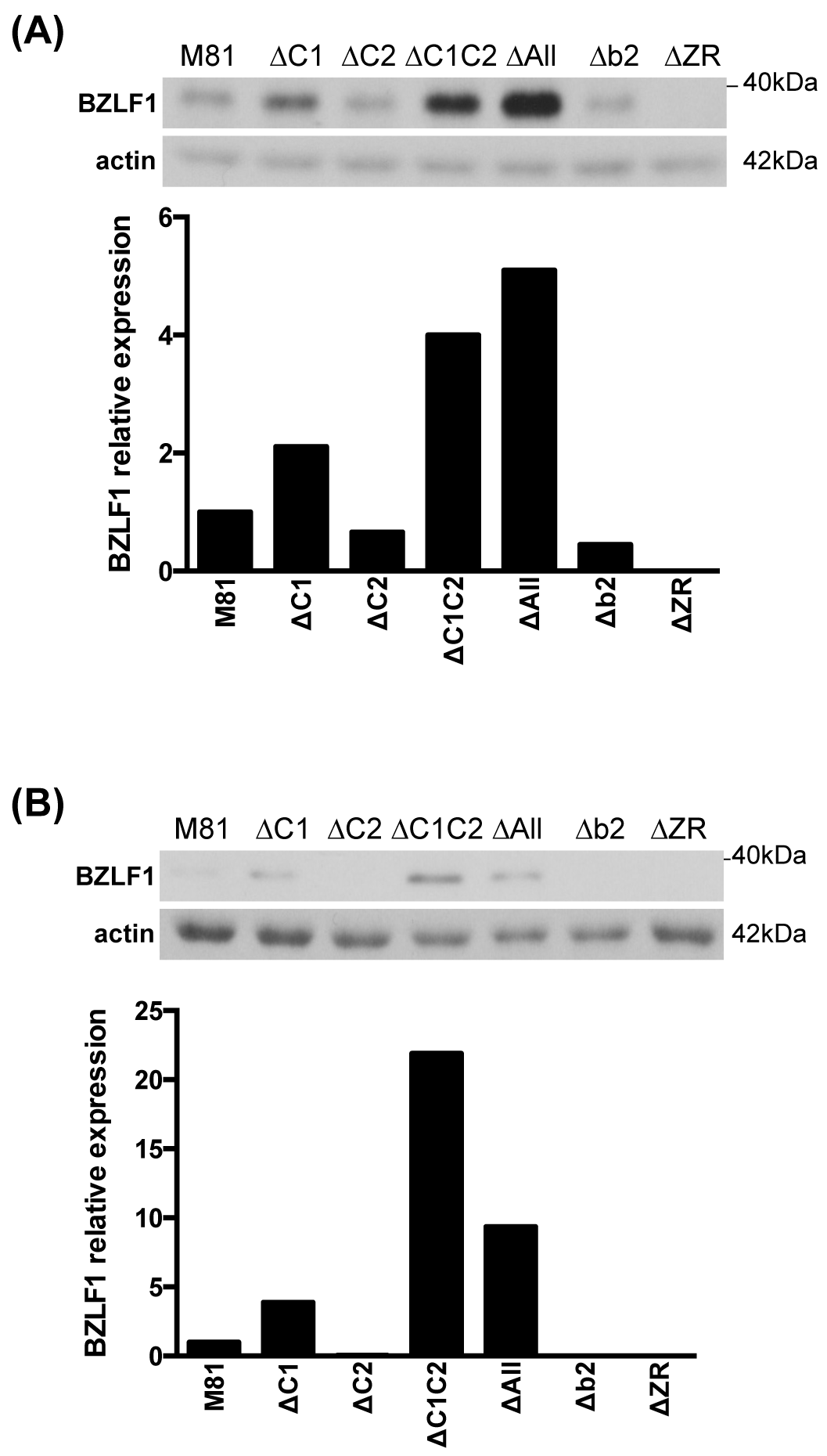

Supplement: S5 Fig — The figure shows Western blot analyses of one set of LCL generated with one B cell sample and M81, M81/ΔAll, M81/ΔC1, M81/ΔC2, M81/ΔC1C2, M81/Δb2, M81/ΔZR. The proteins were stained with a BZLF1-specific antibody. The LCLs were stained at 42 (A) and 101 (B) days post-infection. The relative intensity of the signals were quantified using the ImageJ software and are also displayed as a graph of bars. One more sample is shown in Fig 5. (TIF) [file ppat.1005344.s005.tif]

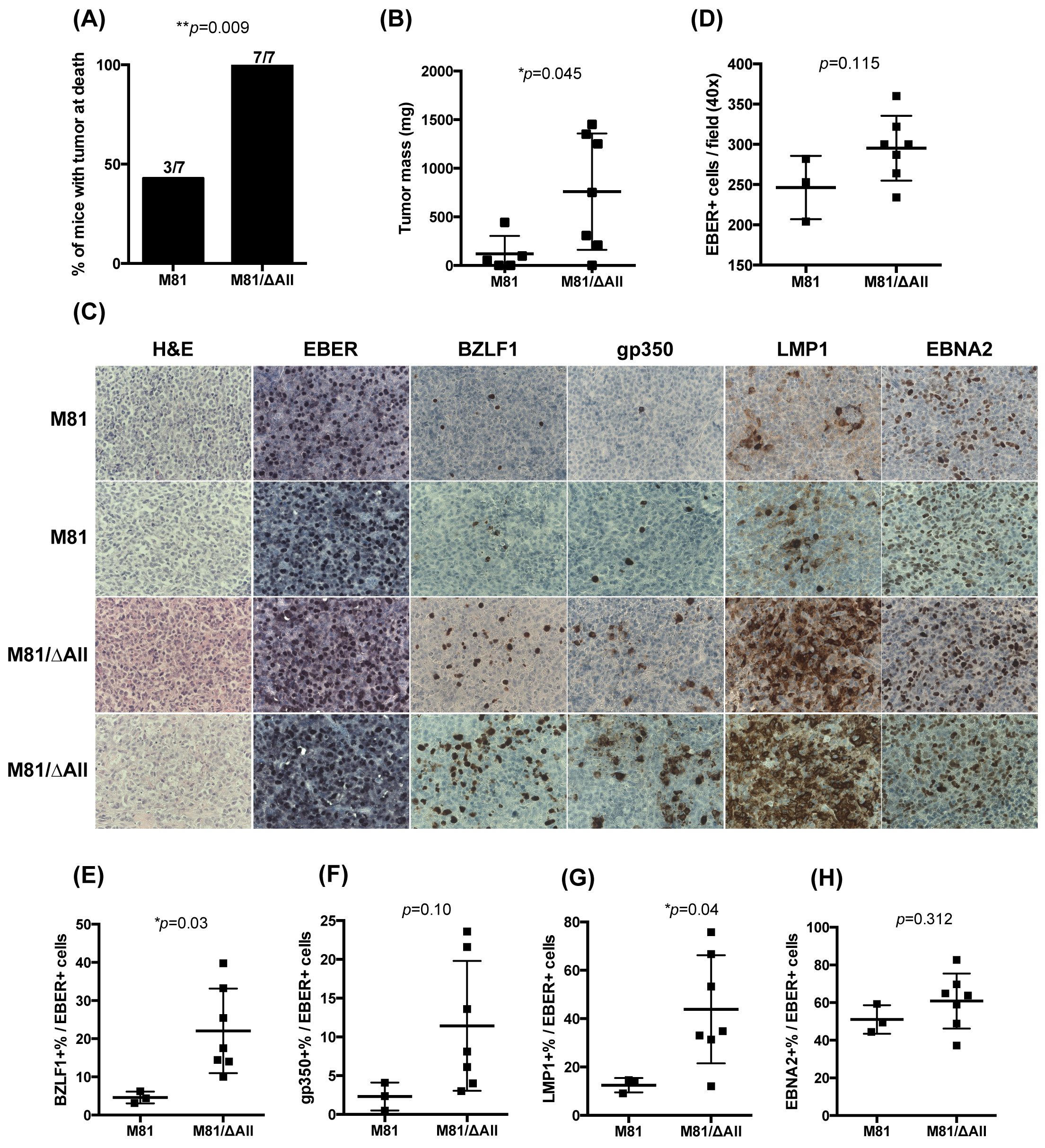

Supplement: S6 Fig — Freshly isolated primary B cells from the peripheral blood were exposed to M81 or M81/ΔAll viruses in vitro for 2 hours at room temperature and immediately injected into NSG mice intraperitoneally. B cells from one blood donor infected with wild type or mutant virus were injected into 8 mice, infected B cells from a second blood donor were injected into 6 mice. The experiment was terminated at the end of the 5th week post-injection. The figure shows the tumor incidence in (A) as well as the weight of the main tumor burden that invaded the pancreatic region in (B). (C-H) show the multiple immunohistological stains of the large tumors that developed in the pancreatic area. (C) Continuous tissue sections were stained with hematoxylin and eosin (H&E), immunostained with antibodies specific for BZLF1, gp350, LMP1, EBNA2, or subjected to an in situ hybridization with an EBER-specific probe. Two tumors from 2 mice in each group are shown. (D) The number of EBER positive cells per 0.04μm2 (surface of the field at high magnification) is given in this boxplot. (E-H) The boxplots display the ratio between (E) BZLF1-, (F) gp350-, (G) LMP1-, or (H) EBNA2-positive cells versus EBER-positive cells. (TIF) [file ppat.1005344.s006.tif]

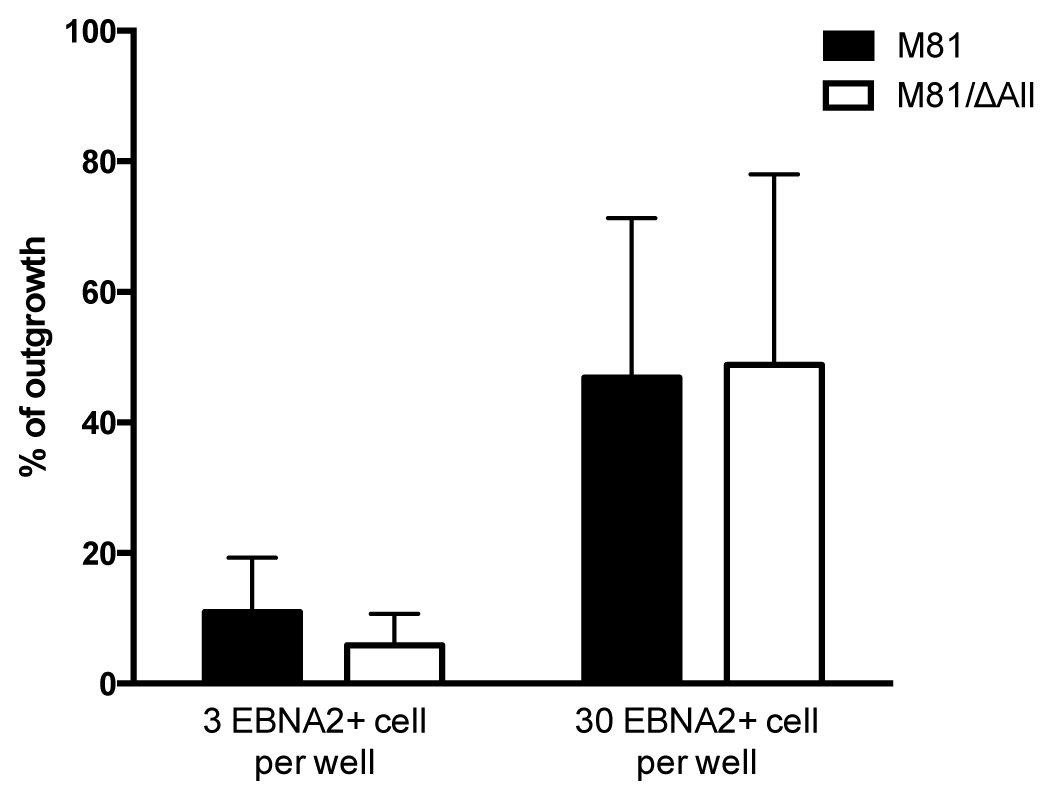

Supplement: S7 Fig — We compared the transformation abilities of M81 and M81/ΔAll by counting the number of outgrowing wells in 96 well plates seeded with infected primary B cells containing either 3 or 30 EBNA2-positive cells after 5 weeks. The bar chart shows the arithmetic mean of four independent experiments and their standard deviation. (TIF) [file ppat.1005344.s007.tif]

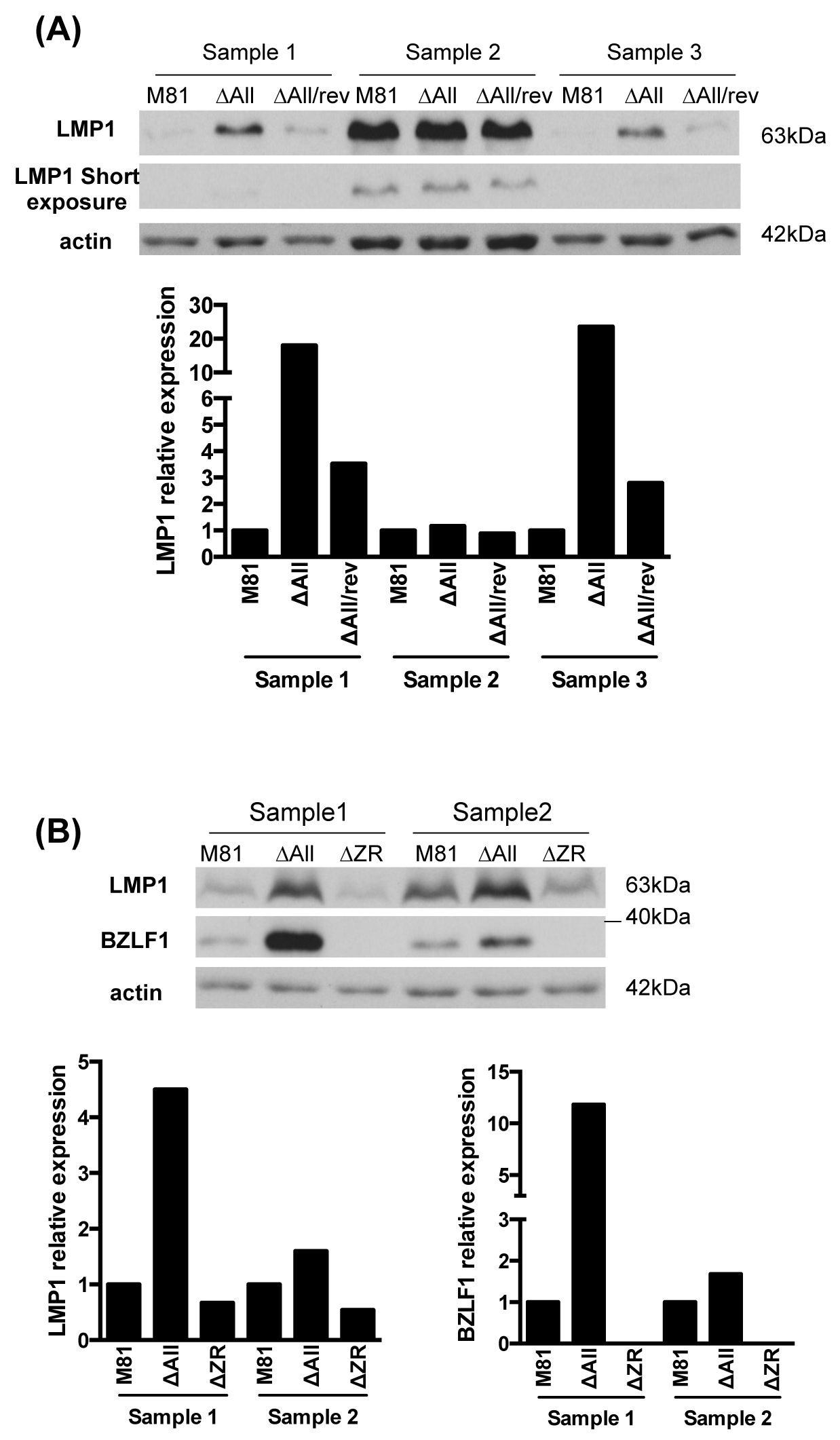

Supplement: S8 Fig — (A) The immunoblots shown in Fig 1A were stripped and restained with antibodies specific for LMP1. (B) Extracts from 2 sets of independent LCLs generated with M81, M81/ΔAll or M81/ΔZR viruses were used to perform Western blots with antibodies against LMP1, BZLF1, and actin. The relative intensity of signals of each analysis was quantified using the ImageJ software and are given as a graph of bars. (TIF) [file ppat.1005344.s008.tif]
