# Supplementary material for: The Epstein-Barr Virus BART miRNA Cluster of the M81 Strain Modulates Multiple Functions in Primary B Cells
Source: PLoS Pathog. 2015 Dec 22;11(12):e1005344. doi: 10.1371/journal.ppat.1005344 (PMC4691206; doi:10.1371/journal.ppat.1005344)
Supplement: S1 Table — (DOCX) [file ppat.1005344.s009.docx]

**S1 Table. Macroscopic and histological features of NSG mice treated with EBV-infected primary B cells.**

| B cell sample | Mouse Tag | EBV+ tumors | | | |
| --- | --- | --- | --- | --- | --- |
|  |  | Pancreas/Gut area | | | Liver |
|  |  | Location | Size of the cut section (mm) | Total mass (mg) | Size of the cut section (mm) |
| 1 | M81-1 | Pancreas/Gut | 8*5 | 98.3 |  |
| 1 | M81-2 | - | - | - |  |
| 1 | M81-3 | Pancreas/Gut | 12*8 | 443 |  |
| 1 | M81-4 | - | - | - |  |
| 2 | M81-1 | - | - | - |  |
| 2 | M81-2 | Pancreas/Gut | 7*4 | 52 |  |
| 2 | M81-3 | - | - | - |  |
| 1 | M81/ΔAll-1 | Pancreas/Gut; infiltration into the kidney | 15*12 | 750 | 2*1; 2*1 |
| 1 | M81/ΔAll-2 | Pancreas/Gut | 20*12 | 1350 | 7*4 |
| 1 | M81/ΔAll-3 | Pancreas/Gut | 19*12 | 1450 | 7*4, 3*2 |
| 1 | M81/∆All-4 | Pancreas/Gut; infiltration into the kidney | 20*12 | 1250 | 3*2 |
| 2 | M81/ΔAll-1 | Pancreas/Gut | 12*10 | 308 |  |
| 2 | M81/ΔAll-2 | Pancreas/Gut | 5*4, 10*5 | 209.8 |  |
| 2 | M81/ΔAll-3 | Pancreas/Gut | 3*2 | 94.2 |  |
|  |  |  |  |  |  |
