# Supplementary material for: The Epstein-Barr Virus BART miRNA Cluster of the M81 Strain Modulates Multiple Functions in Primary B Cells
Source: PLoS Pathog. 2015 Dec 22;11(12):e1005344. doi: 10.1371/journal.ppat.1005344 (PMC4691206; doi:10.1371/journal.ppat.1005344)
Supplement: S2 Table — (DOCX) [file ppat.1005344.s010.docx]

**S2 Table. Oligonucleotides used in this study**

| Name | Sequence^1^ | Aim |
| --- | --- | --- |
| BART3 fwd | 5'-cacaccacctaagaacaaggcatcgttaacctttggtggaaacagctatgaccatgattacgcc-3' | ∆C1 cloning |
| BART6 rev | 5'-tggtgcatttgcaggtgtgaaatgcttggccttgagttacccagtcacgacgttgtaaaacgac-3' |  |
| BART21 fwd | 5'-tagatgttagctttgtgttgggttagtatgggctgggtataacagctatgaccatgattacgcc-3' | ∆C2 cloning |
| BART14 rev | 5'-tgggttggcgtttccgaagagtagcaggtcgcgcgtccagccagtcacgacgttgtaaaacgac-3' |  |
| BART2 fwd | 5'-ggtcctcgaggatgataaacaagatgccagc-3' | ∆b2 cloning |
| BART2 rev | 5'-gcactcgagcgagagaagagagcggcgag-3' |  |
| Overlap fwd | 5'-ctccctgcctggtggacttccagaaacgttgagaaaataaactgtgagtt-3' |  |
| Overlap rev | 5'-aactcacagtttattttctcaacgtttctggaagtccaccaggcagggag-3' |  |
| BXLF1 fwd | 5'-acttccaacaacaccgcgtcggctcggaaaggctgtatgacttccttaagaacagctatgaccatgattacgcc-3' | Cloning of EA-D promoter driven CD2 geen into BXLF1 |
| BXLF1 rev | 5'-ggtcccgggggcagagacaaaaaagaggctagttgctcccagctcttaagccagtcacgacgttgtaaaacgac-3' |  |
| BART3-tet-fwd | 5'-ctgtaaacacacaccacctaagaacaaggcatcgttaacctttggtggaagcggaataacatcatttggtgacg-3' | Cloning of the ∆All revertant |
| BART2-tet-rev | 5'-cccaggggagcgtggcccgtggatctgtgaaactcacagtttattttctcgcgtgtttagattggagtgaacg-3' |  |
| pEP-kans fwd | 5'-tttcatgatatcccgggctgccattattcccttgagtgttatagctaagtagggataacagggtaatcg-3' | Cloning of the ∆All revertant -put I-sceI enzyme restriction sites |
| pEP-kans rev | 5'-tttcatgatatcgccagtgttacaaccaattaacc-3' |  |
| BZLF1 3’UTR fwd | 5'-tcgactcgagcgaggatctcttaaatttctaactcc-3' | PCR/cloning of BZLF1 3’UTR |
| BZLF1 3’UTR rev | 5'-acttgaattccaaagagagccaacaggaag-3' |  |
| BALF5 3’UTR fwd | 5'-tcgactcgagtgagggggcctgagactggaccc-3' | PCR/cloning of BALF5 3’UTR |
| BALF5 3’UTR rev | 5'-agcagaattcggagtaccagacaaaacacgccc-3' |  |
| BART1-3p RT | 5'-ctcaactggtgtcgtggagtcggcaattcagttgagagacatag-3' | Stem-loop RT-qPCR BART 1-3p Taqman PCR |
| BART1-3p probe | 5'FAM-ttcagttgagagacatag-3'TAMRA |  |
| BART1-3p fwd | 5'-acactccagctgggtagcaccgctatccac-3' |  |
| BART1-3p rev | 5'-ctcaactggtgtcgtggagtcggca-3' |  |
| BART7* RT | 5'-ctcaactggtgtcgtggagtcggcaattcagttgagtgtttcat-3' | Stem-loop RT-qPCR BART7* Taqman PCR |
| BART7* probe | 5'FAM-ttcagttgagtgtttcat-3'TAMRA |  |
| BART7* fwd | 5'-acactccagctgggcctggaccttgactat-3' |  |
| BART7* rev | 5'-ctcaactggtgtcgtggagtcggca-3' |  |
| BART2-5p RT | 5'-ctcaactggtgtcgtggagtcggcaattcagttgaggcaagggc-3' | Stem-loop RT-qPCR BART2-5p Taqman PCR |
| BART2-5p probe | 5'FAM-ttcagttgaggcaagggc-tam-3'TAMRA |  |
| BART2-5p fwd | 5'-acactccagctgggtattttctgcattcgc-3' |  |
| BART2-5p rev | 5'-ctcaactggtgtcgtggagtcggca-3' |  |
| BHRF1-1 RT | 5'-ctcaactggtgtcgtggagtcggcaattcagttgagaactccgg-3' | Stem-loop RT-qPCR BHRF1 miR1  Taqman PCR |
| BHRF1-1 probe | 5'FAM-ttcagttgagaactccgg-3'TAMRA |  |
| BHRF1-1 fwd | 5'-acactccagctgggtaacctgatcagcccc-3' |  |
| BHRF1-1 rev | 5'-ctcaactggtgtcgtggagtcggca-3' |  |
| BHRF1-2 RT | 5'-ctcaactggtgtcgtggagtcggcaattcagttgagttcaattt-3' | Stem-loop RT-qPCR BHRF1 miR2  Taqman PCR |
| BHRF1-2 probe | 5'FAM-ttcagttgagttcaattt-3'TAMRA |  |
| BHRF1-2 fwd | 5'-acactccagctgggtatcttttgcggcaga-3' |  |
| BHRF1-2 rev | 5'-ctcaactggtgtcgtggagtcggca-3' |  |
| BHRF1-3 RT | 5'-ctcaactggtgtcgtggagtcggcaattcagttgaggtgtgctt-3' | Stem-loop RT-qPCR BHRF1 miR3  Taqman PCR |
| BHRF1-3 probe | 5'FAM-ttcagttgaggtgtgctt-3'TAMRA |  |
| BHRF1-3 fwd | 5'-acactccagctgggtaacgggaagtgtgta-3' |  |
| BHRF1-3 rev | 5'-ctcaactggtgtcgtggagtcggca-3' |  |
| miR155 RT | 5'-gcccgttaatgctaatcgtgata-3' | Stem-loop RT-qPCR cellular miRNAs  SYBR green |
| miR155 fwd | 5'-gtcgtatccagtgcagggtccgaggtattcgcactggatacgacacccct-3' |  |
| miR155 rev | 5'-gtgcagggtccgag-3' |  |
| miR146 RT | 5'-gtcgtatccagtgcagggtccgaggtattcgcactggatacgacaaccca-3' |  |
| miR146 fwd | 5'-gcccgtgagaactgaattccatg-3' |  |
| miR146 rev | 5'-gtgcagggtccgag-3' |  |
| Let-7i RT | 5'-gtcgtatccagtgcagggtccgaggtattcgcactggatacgacaacagc-3' |  |
| Let-7i fwd | 5'-gcccgtgaggtagtagtttgtgc-3' |  |
| Let-7i rev | 5'-gtgcagggtccgag-3' |  |
| BZLF1 fwd | 5'-acgacgtacaaggaaacc-3' | RT-qPCR for Taqman or SYBR green |
| BZLF1 rev | 5'-cttggcccggcattttct-3' |  |
| BZLF1 probe | 5'FAM-gcattcctccagcgattctggctgta-3'TAMRA |  |
| GADPH fwd | 5'-caatgaccccttcattgacc-3' | SYBR green  RT-qPCR |
| GADPH rev | 5'-tggaagatggtgatgggatt-3' |  |
| BART exon7 RT | 5'-gcctctctgccctgtttgcc-3' (location of M81 genome: 159833-159814) |  |
| BART exon7b fwd | 5'-aggagctgtagaccccgcc-3' (location of M81 genome: 159681-159699) |  |
| BART expn7b rev | 5'-agcgtcgcctggccctggt-3' (location of M81 genome: 159809-159791) |  |
| IPO7 fwd | 5'-tcagtccgataacagtgcttg-3' |  |
| IPO7 rev | 5'-acgatcctttagaactggcag-3' |  |
| HPRT fwd | 5'-tgctgaggatttggaaaggg-3' |  |
| HPRT rev | 5'-acagagggctacaatgtgatg-3' |  |
| LMP1 fwd | 5'-gcacggacaggcattgttc-3' |  |
| LMP1 rev | 5'-aaggccaaaagctgccagat-3' |  |

^1^. Underlined oligonucleotides indicate the homologous regions of EBV for homologous recombination.
